# Stuttering as a spectrum disorder: A hypothesis

**DOI:** 10.1016/j.crneur.2023.100116

**Published:** 2023-11-01

**Authors:** Shahriar SheikhBahaei, Marissa Millwater, Gerald A. Maguire

**Affiliations:** a Neuron-Glia Signaling and Circuits Unit, National Institute of Neurological Disorders and Stroke (NINDS), National Institutes of Health (NIH), Bethesda, 20892, MD, USA; b CenExel Research/ American University of Health Sciences, Signal Hill, CA, 90755, USA

**Keywords:** Childhood-onset fluency disorder, Neurodevelopment, Stuttering, Spectrum disorder

## Abstract

Childhood-onset fluency disorder, commonly referred to as stuttering, affects over 70 million adults worldwide. While stuttering predominantly initiates during childhood and is more prevalent in males, it presents consistent symptoms during conversational speech. Despite these common clinical manifestations, evidence suggests that stuttering, may arise from different etiologies, emphasizing the need for personalized therapy approaches. Current research models often regard the stuttering population as a singular, homogenous group, potentially overlooking the inherent heterogeneity. This perspective consolidates both historical and recent observations to emphasize that stuttering is a heterogeneous condition with diverse causes. As such, it is crucial that both therapeutic research and clinical practices consider the potential for varied etiologies leading to stuttering. Recognizing stuttering as a spectrum disorder embraces its inherent variability, allowing for a more nuanced categorization of individuals based on the underlying causes. This perspective aligns with the principles of precision medicine, advocating for tailored treatments for distinct subgroups of people who stutter, ultimately leading to personalized therapeutic approaches.

## Highlights

• The underlying causes of Childhood-onset fluency disorder (also known as developmental stuttering) are not fully understood.
• Individuals who stutter exhibit diverse etiologies, phenotypes, diagnostic profiles, and treatment responses.
• In this perspective, we argue that stuttering is a heterogeneous condition with likely varied underlying causes.

## 1 Introduction

Childhood-onset fluency disorder (also known as stuttering) as a persistent disruption in the normal fluency and time pattern of speech that is not appropriate for the individual's age (American Psychiatric Association, 2013). Stuttering is the most frequent type of speech disorder and is considered to be a neurodevelopmental motor disorder (Ludlow and Loucks, 2003; Mawson et al., 2016; Smith and Weber, 2016; Watkins et al., 2008). Stuttering occurs in over one percent of adult population worldwide (Yairi and Ambrose, 2013) and can adversely affect the quality of life of those affected. People who stutter may struggle with a lower quality of life, face educational and occupation barriers (Gerlach et al., 2018), and are often faced with limited access to viable treatment (Koedoot et al., 2011). In other neurodevelopmental disorders, such as Tourette Disorder or attention-deficit hyperactivity disorder (ADHD), significant progress has been made in terms of investigating underlying causes, risk factors, diagnosis, and treatments (American Psychiatric Association, 2013; Hyman et al., 2020; Konopka and Roberts, 2016; Muhle et al., 2004). While stuttering has been known for centuries, its etiology is still not fully understood, and no treatments have yet to be approved by the Food and Drug Administration (or other federal government agencies) to address the disfluency of speech.

Stuttering is present in all cultural, racial, ethnic, and economic groups studied (Yairi and Ambrose, 2013); it has been established that stuttering is highly inheritable and genetic factors contribute to more than 80% of cases (Drayna and Kang, 2011; Yairi and Ambrose, 2013). Stuttering is also proposed as a basal ganglia disorder with an association to the elevated levels of presynaptic dopamine (Wu et al., 1995, 1997). In addition, autoimmune reactions could potentially lead to stuttering (Alm, 2020; G. A. Maguire et al., 2010). Therefore, it seems that different conditions may cause the dysfluency phenotype, however, because the underlying etiologies may be different, it is plausible that different treatment plans should be used in each condition to address the disfluency and other co-morbid psychiatric conditions with stuttering.

Since the majority of children who stutter (CWS) recover through an unknown mechanism by their adolescence (Chow and Chang, 2017; Dworzynski et al., 2007; Smith and Weber, 2016; Yairi and Ambrose, 2013), in this perspective, we have mainly focused on adults who stutter (AWS); and, based on inter-individual variability of stuttering phenotypes, imaging data, and responses to treatments/therapies (*see below*), further argued for heterogeneity of stuttering condition. In this context, we propose a new framework for studying and creating personalized treatment plans for AWS. With the availability of more experimental data in CWS and validation of our hypothesis, the proposed framework may prove to be fruitful in understanding and treating children and adolescent stuttering as well.

## 2 Inter-individual variations of stuttering phenotypes

As a multifactorial disorder, stuttering causes functional brain impairments, and therefore, the APA and World Health Organization (WHO) has categorized stuttering under mental and neurodevelopmental disorders (American Psychiatric Association, 2013; World Health Organization, 2015). Due to the dynamic nature of the cognitive, language and motor-control during development, the dysfluency manifests differently with respect to age, i.e., stuttering phenotypes in CWS are different from those in teenagers and AWS (Chang et al., 2008; Einarsdóttir et al., 2020; Hyman et al., 2020; Smith and Weber, 2016, 2017). Moreover, AWS in general show a broad range of symptoms, such as tonic (prolongations) disfluency, clonic (repetitions) disfluency, broken words, blocking, or excess physical tension as well as association with comorbid anxiety and avoidance (American Psychiatric Association, 2013; Bloodstein and Ratner, 2008; Bloodstein, 1960; Blumgart et al., 2010; Craig et al., 2002; Friedlander et al., 2004; Howell, 2007; Iverach et al., 2018; Koedoot et al., 2011; Packman, 2012; Perez and Stoeckle, 2016; Smith et al., 2014; Yang et al., 2017). One of the main manifestations of stuttering is repetition of syllables and these repetitions have different appearances in AWS. While some AWS have difficulties mainly in starting a sentence, others may have involuntary repetitions of other syllables as they speak. Moreover, the severity of the symptoms is not the same between AWS and breathing irregularilities or other involuntary movements such as tremors, eye blinks, and/or tics may be present before or during impairment events (American Psychiatric Association, 2013). In addition, autonomic phenomena such as flushing, perspiration, pallor, and cardiorespiratory events could be present concurrently in some AWS.

It is also important to note that in *some* AWS, avoidance and social anxiety are often the main incapacitating symptoms (Maguire et al., 2020). Although anxiety or emotional disorders do not cause stuttering, in some AWS, stuttering can coexist with these conditions as well as other neuropsychiatric conditions (such as ADHD, obsessive-compulsive disorder [OCD], and social anxiety disorders [SAD]) (Blumgart et al., 2010; Iverach and Rapee, 2014; Iverach et al., 2009; Smith et al., 2014; Yang et al., 2017). It is suggested that co-existence of stuttering and SAD has a greater negative impact on speech fluency in some AWS (Iverach et al., 2018).

In summary, although frequency and severity of stuttering may fluctuate from day to day and with the speaking situation, the existence of broad variations of type and severity of symptoms in AWS (Tichenor and Yaruss, 2019) suggests that stuttering may be a heterogeneous disorder and the phenotypic characteristics may be different from one group to another.

## 3 Imaging studies

It has been more than three decades that imaging techniques such as positron emission tomography (PET), functional magnetic resonance imaging (fMRI), and diffusion tensor imaging (DTI) have been used to investigate and identify brain region(s) that are associated with stuttering. However, these extensive studies have not provided a unified picture of the brain region(s) involved in the pathophysiology of stuttering. For instance, activations of the cerebellar motor cortex (Fox et al., 1996), insula (Fox et al., 1996; Ingham et al., 2003), thalamus (Chang and Guenther, 2019; Fox et al., 1996), globus pallidus of basal ganglia (Fox et al., 1996; Wu et al., 1995), and periaqueductal grey (Braun et al., 1997) were reported in AWS. In other studies, atypical neural activation of the anterior cingulate cortex (Kroll et al., 1997; Pool et al., 1991) as well as disproportionate activation of anterior forebrain regions (cortex, hippocampus, and basal ganglia) or a decrease in activities of superior temporal gyrus are reported (Craig;McQuaide et al., 2014; Démonet et al., 1992; Ingham et al., 2003; Petersen et al., 1988). Moreover, reduced global brain blood flow (Pool et al., 1991) or increase in activities of right caudate of basal ganglia and frontotemporal cortex (Brown et al., 2005; Neef et al., 2018a; Van Borsel et al., 2003) are reported in people with stuttering. In addition, atypical brain activity patterns in the left premotor cortex and posterior auditory cortex are shown in AWS (Braun et al., 1997; Chang and Guenther, 2019; Chang et al., 2009; Fox et al., 1996; Watkins et al., 2008). Moreover, atypical symmetry of the planum temporale (a region in the posterior temporal lobe) across both hemispheres was showed in some people who stutter (Foundas et al., 2001, 2004). Recently, an increase in the volume of the right nucleus accumbens (Neef et al., 2018b) and a decrease in functional connectivity between left inferior frontal gyrus, premotor cortex, supramarginal, and posterior auditory cortices were reported in AWS (Chang et al., 2011; Connally et al., 2014; Lu et al., 2009; Sommer et al., 2002; Watkins et al., 2008).

This heterogeneity in both anatomical and functional human imaging studies further support the hypothesis that stuttering may not be a homogeneous disorder. Therefore, without knowing the underlying etiologies or at least variants in stuttering phenotypes, placing people who stutter in one experimental group, and comparing their imaging data with a control group may not be appropriate and may mask important findings.

## 4 Responses to therapies/treatments

Although several medications (such as risperidone, olanzapine, ecopipam, and others) have been proposed (Maguire et al., 2000a, 2004, 2019, 2020; Shaygannejad et al., 2013), no therapeutic agent has yet been approved by the FDA for the treatment of stuttering, with speech and cognitive therapies remaining first-choice treatments (Brignell et al., 2020; Menzies et al., 2008). While different speech and behavioral therapies (such as direct and indirect Lidcombe program, cognitive behavioral therapy [CBT], etc.) for stuttering have shown narrow significant improvement in controlled clinical trials, they were associated with varying relapse rates, as well as negative effects on the normal pattern of speech (Novelli, 2018). In addition, heterogeneous responses to speech therapy among people with stutter are also reported (Andrews et al., 2012; Baxter et al., 2015; Block et al., 2006; Brignell et al., 2020; O'Brian et al., 2013). Other nonpharmaceutical approaches that are currently being researched are transcranial direct current stimulation (tDCS) and repetitive transcranial magnetic stimulation (rTMS). Recently, tDCS was used in the left inferior frontal cortex to expand behavioral therapy interventions including metronome-timed speech and choral speech (Chesters et al., 2018). rTMS has been used to restructure the temporal integration of intracortical motor circuits in the brain (Busan et al., 2019). However, the fact that even in these limited trials, tDCS and rTMS did not improve fluency of speech in *all* subjects further strengthens the hypothesis that stuttering is not a homogeneous disorder.

Several pharmaceutical agents including dopamine modulators (risperidone, olanzapine, lurasidone, ecopipam, and aripiprazole), GABA enhancers (pagoclone), and others (levetiracetam) showed some improvements in symptoms of stuttering (Charoensook and Maguire, 2017; Ghazavi et al., 2020; Maguire et al., 2000a, 2004, 2019, 2021; G. Maguire et al., 2010; Mozos-Ansorena et al., 2012; Shaygannejad et al., 2013; see Maguire et al. (2020) for a comprehensive review on this topic.) However, similar to heterogeneity in response to speech therapy, such is also observed in the pharmacologic treatment of stuttering in AWS (Ghazavi et al., 2020; Maguire et al., 2019). Although dopamine receptor 2 (D2R) antagonist agents have shown positive results across many formalized trials, no D2R agent has been entered in a modest sized Phase II clinical trial to measure their effect size in a more definitive trial that would have supported a further step to FDA approval. On the other hand, Ecopipam (dopamine receptor 1 antagonist) and Pagoclone (partial GABA-A agonist) worked on different mechanisms from D2R antagonists, and they did not yield as strong as D2R agents in smaller trails; yet various individual self-reported results suggest personal improvement of stuttering from one agent rather than the other ones. Such heterogeneity in response to treatments, similar to other neurodevelopmental conditions, suggests that varying effects of pharmacological treatment are observed across what are thought as a homogenous condition and further support our hypothesis.

## 5 Causes of stuttering disorders

Considering stuttering as a heterogeneous condition suggests that the underlying mechanisms could also be heterogeneous. Recent experimental data point toward involvement of at least three *potential* causes for stuttering. These include specific mutations in genes involved in cellular trafficking, disorders of basal ganglia circuits, and autoimmune reactions. In the next sections, we will discuss each of these three *potential* etiologies of stuttering disorders in more details. In addition to the abovementioned etiologies, recent studies have proposed a connection between elevated iron deposits in the brain (in the basal ganglia and other speech-related circuits) and stuttering (Cler et al., 2021, Liman et al., 2021). However, additional research is needed to determine whether these increased iron levels are indeed a causative factor for stuttering disorders (Sommer et al., 2021).

### 5.1 Lysosomal enzyme-targeting pathway and stuttering

Genetic substrates play a significant role in pathophysiology of stuttering. It has been shown that specific point mutations in four genes that are involved in the lysosomal enzyme-targeting pathway (i.e., *GNPTAB*, *GNPTG*, *NAGPA*, and *AP4E1)* are linked to stuttering disorder (Han et al., 2019; Kang et al., 2010; Lee et al., 2011; Raza et al., 2015), in which they may contribute to up to 20% of the cases (Frigerio;Domingues and Drayna, 2017). One important aspect of this pathway involves addition of mannose -6-phosphate (m6p) to the cargo in two-steps. The first step involves the *N*-acetylglucosamine-1-phosphate transferase enzyme (GlcNAc-phosphotransferase). While GlcNAc-phosphotransferase is made up of alpha (α), beta (β), and gamma (γ) subunits, *GNPTAB* gene produces the alpha and beta subunits, and *GNPTG* produces the gamma subunit (Kudo et al., 2006; Raas-;Rothschild et al., 2000; Tiede et al., 2005, 2004). The second step in lysosomal enzyme-targeting pathway involves N-acetylglucosamine-1-phosphodiester alpha-N-acetylglucosaminidase (*NAGPA*) (Kang et al., 2010). It has been reported that point mutations in highly conserved locations in *GNPTAB*, *GNPTG*, and *NAGPA* genes were linked to stuttering disorders (Kang et al., 2010). Later, it was also shown that mutations in *AP4E1* gene that encode a subunit of the AP-4 complex, which interacts with *NAGPA*, was involved in protein sorting at the trans-Golgi complex, were also linked to developmental disorders (Raza et al., 2015). Interestingly, when in a mouse model carrying a mutation homologue to the human stuttering mutation (*Gnptab-mutant*), the male mice displayed longer pauses and less vocalizations, similar vocalization abnormalities in humans who have this mutation and stutter ( Barnes et al., 2016).

Recent imaging studies in (a group of) people who stutter suggested that alterations in the organization of functional connectivity in brain regions associated with stuttering were spatially colocalized with expression pattern of *GNPTG* in human brain (Benito;Aragón et al., 2020). In addition, the magnitude of differences in grey matter volume between people who stutter and the control group exhibited a strong positive spatial correlation with the expression profile of *GNPTG* and *NAGPA* (Chow et al., 2020). In addition to these anatomical studies, it was also found that a mutation in one of *GNPTAB*, *GNPTG*, *NAGPA*, and *AP4E1* genes was linked to a poorer response to speech therapies in people who stutter (Frigerio;Domingues et al., 2019). Therefore, genetics may play an imperative role in therapy, and therapy outcomes in people who stutter. Consequently, in a sub-group of people who stutter with known genetic mutation, it is plausible that the treatment plan could involve pharmacogenetics or gene replacement therapies.

Recent studies have indicated that mutations in genes associated with the dopaminergic system, specifically *DRD2* and *SLC6A3*, may be linked to stuttering, in addition to genes involved in the lysosomal targeting pathway. (Chen et al., 2014; Han et al., 2014; Lan et al., 2009; Mohammadi et al., 2018; Montag et al., 2012). Moreover, it was recently shown that mutations in *ARNT2*, *SSUH2*, *CYRIA*, *ZMAT4* (and maybe other genes), are also likely linked to stuttering (Below et al., 2023, Polikowsky et al., 2022, Shaw et al., 2021). The fact that these genes do not have a common cellular pathway further supports our hypothesis that the underlying mechanisms of stuttering are different. Further research is essential to better understand the role of specific genes in the development of stuttering disorders. For a recent update on a possible role of the *ARNT2* gene in the pathophysiology of stuttering, see (Alm, 2021).

### 5.2 Dopamine and stuttering

Many lines of evidence suggest that stuttering is related to malfunctions of the basal ganglia circuits, which are important for the initiation of activities in speech motor circuits. The first group of evidence comes from imaging studies that showed stuttering is associated with the elevated levels of presynaptic dopamine (Wu et al., 1995, 1997). This dopamine hypothesis, which was further developed (Alm, 2004, Alm, 2004; Maguire et al., 2002), is supported by recent findings as well as current limited clinical studies (Chang and Guenther, 2019; Maguire et al., 2000a, 2004, 2019, 2020; Shaygannejad et al., 2013). The functions of the basal ganglia significantly rely on the precise and regulated activities of the neurotransmitter dopamine, such that dopaminergic medications have shown to have the strongest effect on stuttering in AWS (Charoensook and Maguire, 2017; Maguire et al., 2000b, 2004, 2019, 2020, 2021). Recently, we have shown that dopamine D2 receptor blockers (such as risperidone) increase activities of the basal ganglia, possibly by activating astrocytes in this region (Maguire et al., 2021; Turk et al., 2021). Surprisingly, in a group of people who stutter, dopamine-stimulant medications (such as methylphenidate) could improve stuttering phenotypes (SheikhBahaei et al., 2022). This report further support that stuttering is a heterogeneous disorder and the underlying mechanisms that lead to stuttering, even those related to dopaminergic circuits, could be diverse.

On the other hand, and consistent with the excess dopamine hypothesis, recent data suggest that, at least in a subgroup of people who stutter, improvement in speech fluency after speech therapy was associated with increased activity of basal ganglia (Giraud et al., 2008; Toyomura et al., 2015). Therefore, we propose that a combination of speech therapy and medical intervention may synergistically reduce speech disfluency in this group of people who stutter who have abnormal basal ganglia function. However, more experiments are needed to delineate the cellular and subcircuit(s) mechanisms that are affected in the basal ganglia, as well as the possible role of astrocytes in the pathogenesis of stuttering.

### 5.3 Autoimmune response and stuttering

Other than genetic factors and excess dopamine, autoimmune reactions could also cause stuttering. It is reported that pediatric autoimmune disorders after an infection with group A beta-hemolytic streptococcus (GAS) can lead to stuttering (Maguire et al., 2010; Alm, 2020). In these conditions, antibodies produced against streptococcal proteins could potentially cross-react with proteins/receptors on the surface of the brain cells (for instance, with dopamine 2 receptors [D2R] in basal ganglia) and potentially disturb the developing speech motor circuits and cause stuttering.

The immune reactions against GAS infections are known to be involved in some neurological symptoms related to the basal ganglia (Swedo and Grant, 2005). For instance, existence of antibodies against the basal ganglia dopamine D2 receptors in patients with Sydenham's chorea (Dale and Brilot, 2012; Dale et al., 2012), a neurological disorder resulting from infection with GAS, further strengthen the hypothesis that autoimmune reaction could lead to stuttering phenotypes. In this subgroup of people who stutter with a history of bacterial infection, it has been shown that antibiotic therapies in patients who stutter resulted in the long-term termination of the stuttering symptoms within 2 weeks (Maguire et al., 2010). This indicates that, for this subgroup, addressing the infection or immune response might suffice, without the need for additional interventions. However, further investigation, potentially using animal models, is necessary to elaborate on this potential cause of stuttering.

## 6 Stuttering as a spectrum disorder

**Fig. 1:**
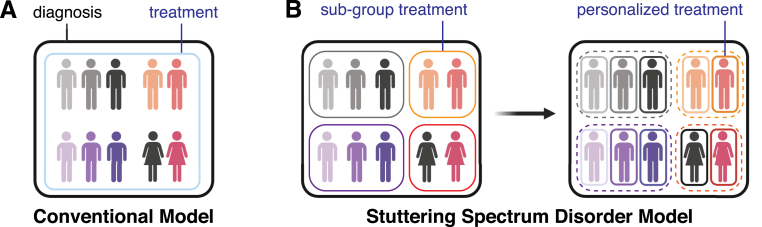
Exploring research **and treatment of stuttering** through **the Stuttering Spectrum Disorder (SSD)** framework**. (A)** Traditional stuttering models facilitate early and effective diagnosis due to the disorder's distinctive phenotype (i.e., stuttering). Yet, by categorizing all individuals who stutter into a singular group (blue line), these models may neglect the inherent diversity in etiology, symptomatology, and treatment responses. **(B)** The proposed Stuttering Spectrum Disorder (SSD) model acknowledges this heterogeneity among adults who stutter (AWS). By stratifying AWS based on genetic and phenotypic variations, the SSD model enhances traditional approaches, enabling subgroup-specific treatments (left). This paradigm aligns with precision medicine's principles (right), aiming for tailored interventions for AWS. Subgroups are depicted in varying color shades. (For interpretation of the references to color in this figure legend, the reader is referred to the Web version of this article.)

Similar to autism spectrum disorder (ASD), ADHD, and other neurodevelopmental disorders, there are no biomarkers to diagnose stuttering, and the diagnosis is usually made by clinical presentations (American Psychiatric Association, 2013). As discussed, existence of several distinct suggested etiologies for stuttering (i.e., genetics, excess dopamine, autoimmune response, and maybe others) strongly point toward presence of subgroups of stuttering disorders (Fig. 1). In fact, the notion of heterogeneity in stuttering is not new and the existence of subgroups in this disorder has been proposed before (Blood, 1985; Brown, 1945; Kell et al., 2009; Riley and Riley, 2000; St Onge and Calvert, 1964; Yairi, 2007; for a comprehensive review on this topic see, Yairi, 2007). Other than the evidence presented in the previous section, other existing data supports the hypothesis that stuttering is a heterogeneous disorder. For instance, phenotypically and based on variety of disfluencies, several differences in people who stutter are reported. For example, differences in repetitions and prolongations of sounds is reported in people who stutter (Schwartz and Conture, 1988). Interestingly, differences in cognition and laryngeal functions are reported between adults who exhibit prolongations or repetitions stuttering (Conture et al., 1977; Feinberg et al., 2000). These data further support the hypothesis that there may be different subgroups among the people who stutter. In addition, subgroups of AWS based on effects of linguistic and demands of motor planning on speech production processes and stuttering has been proposed before (Haj;Tas, 2007; Logan, 2003; Watson et al., 1994). Furthermore, it has been shown that the daily stresses have *different* effects on people who stutter and a subgroup of PWS and perceiving more stresses, speak less fluently (Brantley et al., 1987; Haj-Tas, 2007). In addition, recent reports suggest heterogeneity even in the dopamine hypothesis as well as possible relationships between stuttering with migraine or seizure (Papadopoulou et al., 2022, Wong et al., 2021; SheikhBahaei et al., 2022; Turk et al., 2023). Although more studies are needed to further explore these interesting reports, all together, these data further strengthen the hypothesis that AWS are a heterogeneous group and that generalizations of stuttering behaviors might not always be justifiable. In this context, to better describe this disorder and to account for all the various causes and symptoms associated with it, we propose stuttering to be considered as a spectrum disorder. With improved imaging techniques, next-generation DNA sequencing technologies, advanced electrophysiology, availability of more animal models, and sophisticated computational algorithms, more studies should be done to define characteristics of each subtype of the stuttering under the umbrella of stuttering spectrum disorders (SSD). For instance, combining genomic testing with next-generation analyses of imaging data will help researchers and clinicians to set criteria for each SSD subtype and predict if a person who stutter would respond to certain therapies. In this regard, experiments on animal models would be essential to dissect molecular, cellular, and circuit mechanisms underlying stuttering.

Although the availability of experimental data in CWS is limited relative to AWS, the proposed SSD framework may facilitate current research in the determination of etiologies and treatment paths for children who stutter as well. To date, there are comparatively few neuroimaging studies in CWS due to the practical challenges of neuroimaging in children (such as prolonged restriction of the head). Of the few neuroimaging studies conducted in CWS and their control peers, a range of findings were reported (Chang, 2014). In structural MRI studies, differences were found in white matter integrity and grey matter volume in children who stutter compared to fluent speakers (Beal et al., 2013; Chang et al., 2008). Both persistent stuttering children and recovered groups showed reduced grey matter volume in speech-related regions, while in persistent stuttering children, but not recovered children, reduced fractional anisotropy was found in left white matter tracts underlying motor regions compared to controls (Chang et al., 2008). Asymmetry patterns reported in AWS, when assessed in CWS, yielded conflicting results, with one study reporting no differences and another reporting increased grey matter volume in speech-related structures in the right hemisphere (Chang et al., 2008; Beal et al., 2013) Conflicting findings were also reported regarding white matter volume in the corpus callosum, with one study detecting less white matter volume in children who stutter bilaterally, and another study reporting no differences between children with persistent stuttering, children who recovered from stuttering, and typically fluent children (Beal et al., 2013; Choo et al., 2012).

Functional brain studies in children who stutter have largely employed fMRI. One such study reported nonstuttering speakers to exhibit left laterality of brain response in linguistic and prosodic brain functions, whereas stuttering peers showed no left laterality (Sato et al., 2011). In another fMRI study, CWS were seen to have reduced functional connectivity in regions that support the timing of movement (Chang and Zhu, 2013). Ultimately, the range of findings in neuroimaging studies of CWS supports that the proposed framework be used to inform future study development, particularly to define experimental groups that account for the heterogeneity in origin and/or phenotype of stuttering.

It has been previously noted that behavioral and speech language therapies are the current line of defense for CWS. In a review of empirical evidence on intervention approaches designed to address childhood stuttering, the most effective delivery reduced stuttering severity from a baseline of 3.8–9.4% to 0.9–3.7% post-intervention (Brignell et al., 2021)(). Interestingly, the likelihood of a child who stutters to recover, relapse, or persistent in stuttering may be tied to underlying genetic factors transmitted in addition to the underlying susceptibility to stuttering (Ambrose et al., 1997). Better understanding the way in which genetic factors are involved in the development and/or persistence of stuttering disorders might allow for accurate evaluation and development of therapies for CWS.

## 7 Conclusions and future perspectives

If our hypothesis proved to be correct, SSD could refer to a broad range of conditions characterized by challenges with speech communication (i.e., stuttering phenotype). Future investigations are essential to research the etiology and expand on how identified genetic substrates alter the activity or synchronization of brain circuits that eventually lead to stuttering behaviors. In this regard, finding the cellular and circuit mechanisms underlying stuttering will lead to identification of novel therapeutic agents for more effective treatments. These studies might eventually lead to an integrated model to study and treat other forms of speech disorders (such as, adult-onset fluency disorder). Under SSD framework, each person with stuttering has a distinct set of strengths and challenges, and individuals who stutter are empowered to have a freedom of choice to pursue their own personalized treatment course.

Upon availability of more experimental data (for instance, a combination of genetics data with longitudinal imaging data in CWS) and validation of our hypothesis, it may be possible to expand the SSD framework to include children who stutter. With the advancement of imaging techniques, sophisticated genetic analysis, and the increasing availability of diverse animal models, we are now poised to accurately delineate subtypes of stuttering disorders. Identifying novel biomarkers will be pivotal in predicting which individuals belong to a specific stuttering subtype and determining the most effective treatment plan for them. Indeed, research into identifying subtypes in other neurodevelopmental disorders has gained significant attention in recent years. Similarly, we believe SSD has the potential to deconstruct stuttering heterogeneity into homogeneous subtypes. Discovering the subtypes of neural circuits implicated in SSD may open new avenues for innovative treatment methods. These could include targeted gene- or cell-replacement therapies, pharmacogenetic strategies, or non-invasive devices complementing traditional speech and cognitive treatment plans. Consequently, personalized therapy designed for particular etiological subtypes might become crucial in the near future for optimizing clinical treatments and improving the quality of life for individuals who stutter.

## Author contributors

S.SB, M.M, and G.A.M prepared the manuscript. All authors approved the final version of the manuscript.

## Funding sources

This work was supported by the Intramural Research Program of the 10.13039/100000002NIH, 10.13039/100000065NINDS (ZIA NS009420 to S.SB.).

## Declaration of competing interest

The authors declare the following financial interests/personal relationships which may be considered as potential competing interests: GAM currently has research grants from Aptinyx, Cerevel, Compass, Emalex, IntraCellular, Jazz, Karuna, Merck, Neurocrine, Noema, Otsuka, Sunovion, and Teva; has consultant agreements with Biogen, Emalex, Karuna, Noema, and Sage; and is a speaker for Alkermes, Axsome, Janssen, and Otsuka. . Other authors declare no conflict of interest.

## Data Availability

No data was used for the research described in the article.

## References

[bib1] Alm P.A. (2004). Stuttering and the basal ganglia circuits: a critical review of possible relations. J. Commun. Disord..

[bib2] Alm P.A. (2021). Stuttering: a disorder of energy supply to neurons?. Front. Hum. Neurosci..

[bib3] Alm P.A. (2020). Streptococcal infection as a major historical cause of stuttering: data, mechanisms, and current importance. Front. Hum. Neurosci..

[bib4] Alm P.A. (2004). Stuttering and the basal ganglia circuits: a critical review of possible relations. J. Commun. Disord..

[bib138] Ambrose N.G., Cox N.J., Yairi E. (1997). The genetic basis of persistence and recovery in stuttering. *Journal of speech, language, and hearing research*. JSLHR.

[bib5] American Psychiatric Association (2013).

[bib6] Andrews C., O'Brian S., Harrison E., Onslow M., Packman A., Menzies R. (2012). Syllable-timed speech treatment for school-age children who stutter: a phase I trial. Lang. Speech Hear. Serv. Sch..

[bib137] Barnes TD, Wozniak DF, Gutierrez J, Han T-U, Drayna D, Holy TE (2016). A Mutation Associated with Stuttering Alters Mouse Pup Ultrasonic Vocalizations. Curr Biol. April.

[bib7] Baxter S., Johnson M., Blank L., Cantrell A., Brumfitt S., Enderby P., Goyder E. (2015). The state of the art in non-pharmacological interventions for developmental stuttering. Part 1: a systematic review of effectiveness. Int. J. Lang. Commun. Disord.

[bib8] Beal D.S., Gracco V.L., Brettschneider J., Kroll R.M., De Nil L.F. (2013). A voxel-based morphometry (VBM) analysis of regional grey and white matter volume abnormalities within the speech production network of children who stutter. Cortex.

[bib133] Below J., Polikowsky H., Scartozzi A., Shaw D., Pruett D., Chen H.-H., Petty L., Petty A., Lowther E., Yu Y., Research Team, Highland H., Avery C., Harris K.M., Gordon R., Beilby J., Viljoen K., Jones R., Huff C., Kraft S.J. (2023). Discovery of 36 loci significantly associated with stuttering. Res. Sq..

[bib9] Benito-Aragón C., Gonzalez-Sarmiento R., Liddell T., Diez I., d'Oleire Uquillas F., Ortiz-Terán L., Bueichekú E., Chow H.M., Chang S.-E., Sepulcre J. (2020). Neurofilament-lysosomal genetic intersections in the cortical network of stuttering. Prog. Neurobiol..

[bib10] Block S., Onslow M., Packman A., Dacakis G. (2006). Connecting stuttering management and measurement: IV. Predictors of outcome for a behavioural treatment for stuttering. Int. J. Lang. Commun. Disord.

[bib11] Bloodstein O., Ratner N.B. (2008).

[bib12] Bloodstein O. (1960). The development of stuttering. I. Changes in nine basic features. J. Speech Hear. Disord..

[bib13] Blood G.W. (1985). Laterality differences in child stutterers: heterogeneity, severity levels, and statistical treatments. J. Speech Hear. Disord..

[bib14] Blumgart E., Tran Y., Craig A. (2010). Social anxiety disorder in adults who stutter. Depress. Anxiety.

[bib15] Brantley P.J., Waggoner C.D., Jones G.N., Rappaport N.B. (1987). A Daily Stress Inventory: development, reliability, and validity. J. Behav. Med..

[bib16] Braun A.R., Varga M., Stager S., Schulz G., Selbie S., Maisog J.M., Carson R.E., Ludlow C.L. (1997). Altered patterns of cerebral activity during speech and language production in developmental stuttering. An H2(15)O positron emission tomography study. Brain.

[bib17] Brignell A., Krahe M., Downes M., Kefalianos E., Reilly S., Morgan A.T. (2020). A systematic review of interventions for adults who stutter. J. Fluen. Disord..

[bib18] Brown S., Ingham R.J., Ingham J.C., Laird A.R., Fox P.T. (2005). Stuttered and fluent speech production: an ALE meta-analysis of functional neuroimaging studies. Hum. Brain Mapp..

[bib126] Brignell A., Krahe M., Downes M., Kefalianos E., Reilly S., Morgan A. (2021). Interventions for children and adolescents who stutter: A systematic review, meta-analysis, and evidence map. Journal of fluency disorders.

[bib19] Brown S.F. (1945). The loci of stutterings in the speech sequence. J. Speech Disord..

[bib20] Busan P., Del Ben G., Russo L.R., Bernardini S., Natarelli G., Arcara G., Manganotti P., Battaglini P.P. (2019). Stuttering as a matter of delay in neural activation: a combined TMS/EEG study. Clin. Neurophysiol..

[bib21] Chang S.-E., Erickson K.I., Ambrose N.G., Hasegawa-Johnson M.A., Ludlow C.L. (2008). Brain anatomy differences in childhood stuttering. Neuroimage.

[bib22] Chang S.-E., Guenther F.H. (2019). Involvement of the cortico-basal ganglia-thalamocortical loop in developmental stuttering. Front. Psychol..

[bib23] Chang S.-E., Horwitz B., Ostuni J., Reynolds R., Ludlow C.L. (2011). Evidence of left inferior frontal-premotor structural and functional connectivity deficits in adults who stutter. Cerebr. Cortex.

[bib24] Chang S.-E., Kenney M.K., Loucks T.M.J., Ludlow C.L. (2009). Brain activation abnormalities during speech and non-speech in stuttering speakers. Neuroimage.

[bib25] Chang S.-E., Zhu D.C. (2013). Neural network connectivity differences in children who stutter. Brain.

[bib26] Chang S.-E. (2014). Research updates in neuroimaging studies of children who stutter. Semin. Speech Lang..

[bib27] Charoensook J., Maguire G.A. (2017). A case series on the effectiveness of lurasidone in patients with stuttering. Ann. Clin. Psychiatr..

[bib28] Chen H., Wang G., Xia J., Zhou Y., Gao Y., Xu J., Huen M.S., Siok W.T., Jiang Y., Tan L.H., Sun Y. (2014). Stuttering candidate genes DRD2 but not SLC6A3 is associated with developmental dyslexia in Chinese population. Behav. Brain Funct..

[bib29] Chesters J., Möttönen R., Watkins K.E. (2018). Transcranial direct current stimulation over left inferior frontal cortex improves speech fluency in adults who stutter. Brain.

[bib30] Choo A.L., Chang S.-E., Zengin-Bolatkale H., Ambrose N.G., Loucks T.M. (2012). Corpus callosum morphology in children who stutter. J. Commun. Disord..

[bib31] Chow H.M., Chang S.-E. (2017). White matter developmental trajectories associated with persistence and recovery of childhood stuttering. Hum. Brain Mapp..

[bib32] Chow H.M., Garnett E.O., Li H., Etchell A., Sepulcre J., Drayna D., Chugani D., Chang S.-E. (2020). Linking lysosomal enzyme targeting genes and energy metabolism with altered gray matter volume in children with persistent stuttering. Neurobiol. Lang. (Camb).

[bib136] Cler GJ, Krishnan S, Papp D, Wiltshire CEE, Chesters J, Watkins KE (2021). Elevated iron concentration in putamen and cortical speech motor network in developmental stuttering. Brain.

[bib33] Connally E.L., Ward D., Howell P., Watkins K.E. (2014). Disrupted white matter in language and motor tracts in developmental stuttering. Brain Lang..

[bib34] Conture E.G., McCall G.N., Brewer D.W. (1977). Laryngeal behavior during stuttering. J. Speech Hear. Res..

[bib35] Craig-McQuaide A., Akram H., Zrinzo L., Tripoliti E. (2014). A review of brain circuitries involved in stuttering. Front. Hum. Neurosci..

[bib36] Craig A., Hancock K., Tran Y., Craig M., Peters K. (2002). Epidemiology of stuttering in the community across the entire life span. J. Speech Lang. Hear. Res..

[bib38] Dale R.C., Brilot F. (2012). Autoimmune basal ganglia disorders. J. Child Neurol..

[bib39] Dale R.C., Merheb V., Pillai S., Wang D., Cantrill L., Murphy T.K., Ben-Pazi H., Varadkar S., Aumann T.D., Horne M.K., Church A.J., Fath T., Brilot F. (2012). Antibodies to surface dopamine-2 receptor in autoimmune movement and psychiatric disorders. Brain.

[bib40] Démonet J.F., Chollet F., Ramsay S., Cardebat D., Nespoulous J.L., Wise R., Rascol A., Frackowiak R. (1992). The anatomy of phonological and semantic processing in normal subjects. Brain.

[bib41] Drayna D., Kang C. (2011). Genetic approaches to understanding the causes of stuttering. J. Neurodev. Disord..

[bib42] Dworzynski K., Remington A., Rijsdijk F., Howell P., Plomin R. (2007). Genetic etiology in cases of recovered and persistent stuttering in an unselected, longitudinal sample of young twins. Am. J. Speech Lang. Pathol.

[bib43] Einarsdóttir J.T., Crowe K., Kristinsson S.H., Másdóttir T. (2020). The recovery rate of early stuttering. J. Fluen. Disord..

[bib44] Feinberg A.Y., Griffin B.P., Levey M. (2000). Psychological aspects of chronic tonic and clonic stuttering: suggested therapeutic approaches. Am. J. Orthopsychiatry.

[bib45] Foundas A.L., Bollich A.M., Corey D.M., Hurley M., Heilman K.M. (2001). Anomalous anatomy of speech-language areas in adults with persistent developmental stuttering. Neurology.

[bib46] Foundas A.L., Bollich A.M., Feldman J., Corey D.M., Hurley M., Lemen L.C., Heilman K.M. (2004). Aberrant auditory processing and atypical planum temporale in developmental stuttering. Neurology.

[bib47] Fox P.T., Ingham R.J., Ingham J.C., Hirsch T.B., Downs J.H., Martin C., Jerabek P., Glass T., Lancaster J.L. (1996). A PET study of the neural systems of stuttering. Nature.

[bib48] Friedlander A.H., Noffsinger D., Mendez M.F., Yagiela J.A. (2004). Developmental stuttering: manifestations, treatment and dental implications. Spec. Care Dent..

[bib49] Frigerio-Domingues C., Drayna D. (2017). Genetic contributions to stuttering: the current evidence. Mol. Genet. Genomic Med..

[bib50] Frigerio-Domingues C.E., Gkalitsiou Z., Zezinka A., Sainz E., Gutierrez J., Byrd C., Webster R., Drayna D. (2019). Genetic factors and therapy outcomes in persistent developmental stuttering. J. Commun. Disord..

[bib51] Gerlach H., Totty E., Subramanian A., Zebrowski P. (2018). Stuttering and labor market outcomes in the United States. J. Speech Lang. Hear. Res..

[bib52] Ghazavi M., Rastgu F., Nasiri J., Yaghini O. (2020). Efficacy of levetiracetam in treatment of childhood stuttering. Int. J. Prev. Med..

[bib53] Giraud A.-L., Neumann K., Bachoud-Levi A.-C., von Gudenberg A.W., Euler H.A., Lanfermann H., Preibisch C. (2008). Severity of dysfluency correlates with basal ganglia activity in persistent developmental stuttering. Brain Lang..

[bib54] Haj-Tas M. (2007).

[bib55] Han T.-U., Park J., Domingues C.F., Moretti-Ferreira D., Paris E., Sainz E., Gutierrez J., Drayna D. (2014). A study of the role of the FOXP2 and CNTNAP2 genes in persistent developmental stuttering. Neurobiol. Dis..

[bib56] Han T.-U., Root J., Reyes L.D., Huchinson E.B., Hoffmann J. du, Lee W.-S., Barnes T.D., Drayna D. (2019). Human GNPTAB stuttering mutations engineered into mice cause vocalization deficits and astrocyte pathology in the corpus callosum. Proc. Natl. Acad. Sci. U.S.A..

[bib57] Howell P. (2007). Signs of developmental stuttering up to age eight and at 12 plus. Clin. Psychol. Rev..

[bib58] Hyman S.L., Levy S.E., Myers S.M. (2020). Council on children with disabilities, section on developmental and behavioral pediatrics. Identification, Evaluation Manag. Children with Autism Spectrum Disord. Pediatr..

[bib59] Ingham R.J., Ingham J.C., Finn P., Fox P.T. (2003). Towards a functional neural systems model of developmental stuttering. J. Fluen. Disord..

[bib60] Iverach L., Jones M., Lowe R., O'Brian S., Menzies R.G., Packman A., Onslow M. (2018). Comparison of adults who stutter with and without social anxiety disorder. J. Fluen. Disord..

[bib61] Iverach L., Jones M., O'Brian S., Block S., Lincoln M., Harrison E., Hewat S., Menzies R.G., Packman A., Onslow M. (2009). Screening for personality disorders among adults seeking speech treatment for stuttering. J. Fluen. Disord..

[bib62] Iverach L., Rapee R.M. (2014). Social anxiety disorder and stuttering: current status and future directions. J. Fluen. Disord..

[bib63] Kang C., Riazuddin S., Mundorff J., Krasnewich D., Friedman P., Mullikin J.C., Drayna D. (2010). Mutations in the lysosomal enzyme-targeting pathway and persistent stuttering. N. Engl. J. Med..

[bib64] Kell C.A., Neumann K., von Kriegstein K., Posenenske C., von Gudenberg A.W., Euler H., Giraud A.-L. (2009). How the brain repairs stuttering. Brain.

[bib65] Koedoot C., Bouwmans C., Franken M.-C., Stolk E. (2011). Quality of life in adults who stutter. J. Commun. Disord..

[bib66] Konopka G., Roberts T.F. (2016). Animal models of speech and vocal communication deficits associated with psychiatric disorders. Biol. Psychiatr..

[bib67] Kroll R., De Nil L., Houle S. (1997). The use of positron emission tomography for the investigation of changes in brain activation patterns following intensive stuttering treatment. J. Fluen. Disord..

[bib68] Kudo M., Brem M.S., Canfield W.M. (2006). Mucolipidosis II (I-cell disease) and mucolipidosis IIIA (classical pseudo-hurler polydystrophy) are caused by mutations in the GlcNAc-phosphotransferase alpha/beta -subunits precursor gene. Am. J. Hum. Genet..

[bib69] Lan J., Song M., Pan C., Zhuang G., Wang Y., Ma W., Chu Q., Lai Q., Xu F., Li Y., Liu L., Wang W. (2009). Association between dopaminergic genes (SLC6A3 and DRD2) and stuttering among Han Chinese. J. Hum. Genet..

[bib70] Lee W.-S., Kang C., Drayna D., Kornfeld S. (2011). Analysis of mannose 6-phosphate uncovering enzyme mutations associated with persistent stuttering. J. Biol. Chem..

[bib135] Liman J, Wolff von Gudenberg A, Baehr M, Paulus W, Neef NE, Sommer M (2021). Enlarged area of mesencephalic iron deposits in adults who stutter. Front Hum Neurosci.

[bib71] Logan K.J. (2003). The effect of syntactic structure upon speech initiation times of stuttering and nonstuttering speakers. J. Fluen. Disord..

[bib72] Ludlow C.L., Loucks T. (2003). Stuttering: a dynamic motor control disorder. J. Fluen. Disord..

[bib73] Lu C., Ning N., Peng D., Ding G., Li K., Yang Y., Lin C. (2009). The role of large-scale neural interactions for developmental stuttering. Neuroscience.

[bib74] Maguire G., Franklin D., Vatakis N.G., Morgenshtern E., Denko T., Yaruss J.S., Spotts C., Davis L., Davis A., Fox P., Soni P., Blomgren M., Silverman A., Riley G. (2010). Exploratory randomized clinical study of pagoclone in persistent developmental stuttering: the EXamining Pagoclone for peRsistent dEvelopmental Stuttering Study. J. Clin. Psychopharmacol..

[bib75] Maguire G.A., LaSalle L., Hoffmeyer D., Nelson M., Lochhead J.D., Davis K., Burris A., Yaruss J.S. (2019). Ecopipam as a pharmacologic treatment of stuttering. Ann. Clin. Psychiatr..

[bib76] Maguire G.A., Nguyen D.L., Simonson K.C., Kurz T.L. (2020). The pharmacologic treatment of stuttering and its neuropharmacologic basis. Front. Neurosci..

[bib77] Maguire G.A., Riley G.D., Franklin D.L., Gottschalk L.A. (2000). Risperidone for the treatment of stuttering. J. Clin. Psychopharmacol..

[bib78] Maguire G.A., Riley G.D., Franklin D.L., Maguire M.E., Nguyen C.T., Brojeni P.H. (2004). Olanzapine in the treatment of developmental stuttering: a double-blind, placebo-controlled trial. Ann. Clin. Psychiatr..

[bib79] Maguire G.A., Riley G.D., Franklin D.L., Wu J.C., Ortiz T. (2000). The dopamine hypothesis of stuttering and its treatment implications. Int. J. Neuropsychopharmacol..

[bib80] Maguire G.A., Riley G.D., Yu B.P. (2002). A neurological basis of stuttering?. Lancet Neurol..

[bib81] Maguire G.A., Viele S.N., Agarwal S., Handler E., Franklin D. (2010). Stuttering onset associated with streptococcal infection: a case suggesting stuttering as PANDAS. Ann. Clin. Psychiatr..

[bib82] Maguire G.A., Yoo B.R., SheikhBahaei S. (2021). Investigation of risperidone treatment associated with enhanced brain activity in patients who stutter. Front. Neurosci..

[bib83] Mawson A.R., Radford N.T., Jacob B. (2016). Toward a theory of stuttering. Eur. Neurol..

[bib84] Menzies R.G., O'Brian S., Onslow M., Packman A., St Clare T., Block S. (2008). An experimental clinical trial of a cognitive-behavior therapy package for chronic stuttering. J. Speech Lang. Hear. Res..

[bib85] Mohammadi H., Joghataei M.T., Rahimi Z., Faghihi F., Farhangdoost H. (2018). Relationship between serum homovanillic acid, DRD2 C957T (rs6277), and hDAT A559V (rs28364997) polymorphisms and developmental stuttering. J. Commun. Disord..

[bib86] Montag C., Bleek B., Faber J., Reuter M. (2012). The role of the DRD2 C957T polymorphism in neuroticism in persons who stutter and healthy controls. Neuroreport.

[bib87] Mozos-Ansorena A., Pérez-García M., Portela-Traba B., Tabernero-Lado A., Pérez-Pérez J. (2012). Stuttering treated with olanzapine: a case report. Actas Esp. Psiquiatr..

[bib88] Muhle R., Trentacoste S.V., Rapin I. (2004). The genetics of autism. Pediatrics.

[bib89] Neef N.E., Anwander A., Bütfering C., Schmidt-Samoa C., Friederici A.D., Paulus W., Sommer M. (2018). Structural connectivity of right frontal hyperactive areas scales with stuttering severity. Brain.

[bib90] Neef N.E., Bütfering C., Auer T., Metzger F.L., Euler H.A., Frahm J., Paulus W., Sommer M. (2018). Altered morphology of the nucleus accumbens in persistent developmental stuttering. J. Fluen. Disord..

[bib91] Novelli A. (2018).

[bib92] O'Brian S., Iverach L., Jones M., Onslow M., Packman A., Menzies R. (2013). Effectiveness of the Lidcombe Program for early stuttering in Australian community clinics. Int. J. Speech Lang. Pathol..

[bib93] Packman A. (2012). Theory and therapy in stuttering: a complex relationship. J. Fluen. Disord..

[bib130] Papadopoulou S, Pavlidou E, Argyris G, Flouda T, Koukoutsidi P, Krikonis K, Shah S, Chirosca-Vasileiou D, Boussios S (2022). Epilepsy and Diagnostic Dilemmas: The Role of Language and Speech-Related Seizures. Journal of Personalized Medicine.

[bib94] Perez H.R., Stoeckle J.H. (2016). Stuttering: clinical and research update. Can. Fam. Physician.

[bib95] Petersen S.E., Fox P.T., Posner M.I., Mintun M., Raichle M.E. (1988). Positron emission tomographic studies of the cortical anatomy of single-word processing. Nature.

[bib132] Polikowsky H.G., Shaw D.M., Petty L.E., Chen H.-H., Pruett D.G., Linklater J.P., Viljoen K.Z., Beilby J.M., Highland H.M., Levitt B., Avery C.L., Mullan Harris K., Jones R.M., Below J.E., Kraft S.J. (2022). Population-based genetic effects for developmental stuttering. HGG Adv.

[bib96] Pool K.D., Devous M.D., Freeman F.J., Watson B.C., Finitzo T. (1991). Regional cerebral blood flow in developmental stutterers. Arch. Neurol..

[bib97] Raas-Rothschild A., Cormier-Daire V., Bao M., Genin E., Salomon R., Brewer K., Zeigler M., Mandel H., Toth S., Roe B., Munnich A., Canfield W.M. (2000). Molecular basis of variant pseudo-hurler polydystrophy (mucolipidosis IIIC). J. Clin. Invest..

[bib98] Raza M.H., Mattera R., Morell R., Sainz E., Rahn R., Gutierrez J., Paris E., Root J., Solomon B., Brewer C., Basra M.A.R., Khan S., Riazuddin S., Braun A., Bonifacino J.S., Drayna D. (2015). Association between rare variants in AP4E1, a component of intracellular trafficking, and persistent stuttering. Am. J. Hum. Genet..

[bib99] Riley J., Riley J.G. (2000). A revised component model for diagnosing and treating children who stutter. CICSD.

[bib100] Schwartz H.D., Conture E.G. (1988). Subgrouping young stutterers: preliminary behavioral observations. J. Speech Hear. Res..

[bib131] Shaw D.M., Polikowsky H.P., Pruett D.G., Chen H.-H., Petty L.E., Viljoen K.Z., Beilby J.M., Jones R.M., Kraft S.J., Below J.E. (2021). Phenome risk classification enables phenotypic imputation and gene discovery in developmental stuttering. Am. J. Hum. Genet..

[bib101] Shaygannejad V., Khatoonabadi S.A., Shafiei B., Ghasemi M., Fatehi F., Meamar R., Dehghani L. (2013). Olanzapine versus haloperidol: which can control stuttering better?. Int. J. Prev. Med..

[bib102] SheikhBahaei S., Farhan M., Maguire G.A. (2022). Improvement of stuttering after administration of methylphenidate - a case report. Personalized Med. Psychiatr..

[bib103] Smith A., Weber C. (2017). How stuttering develops: the multifactorial dynamic pathways theory. J. Speech Lang. Hear. Res..

[bib104] Smith A., Weber C. (2016). Childhood stuttering: where are we and where are we going?. Semin. Speech Lang..

[bib105] Smith K.A., Iverach L., O'Brian S., Kefalianos E., Reilly S. (2014). Anxiety of children and adolescents who stutter: a review. J. Fluen. Disord..

[bib106] Sommer M., Koch M.A., Paulus W., Weiller C., Büchel C. (2002). Disconnection of speech-relevant brain areas in persistent developmental stuttering. Lancet.

[bib125] Sommer M., SheikhBahaei S., Maguire G.A. (2021). An unexpected iron in the fire of speech production. Brain.

[bib107] St Onge K.R., Calvert J.J. (1964). Stuttering research. Q. J. Speech.

[bib108] Swedo S.E., Grant P.J. (2005). Annotation: pandas: a model for human autoimmune disease. JCPP (J. Child Psychol. Psychiatry).

[bib109] Tichenor S.E., Yaruss J.S. (2019). Stuttering as defined by adults who stutter. J. Speech Lang. Hear. Res..

[bib110] Tiede S., Cantz M., Raas-Rothschild A., Muschol N., Bürger F., Ullrich K., Braulke T. (2004). A novel mutation in UDP-N-acetylglucosamine-1-phosphotransferase gamma subunit (GNPTAG) in two siblings with mucolipidosis type III alters a used glycosylation site. Hum. Mutat..

[bib111] Tiede S., Storch S., Lübke T., Henrissat B., Bargal R., Raas-Rothschild A., Braulke T. (2005). Mucolipidosis II is caused by mutations in GNPTA encoding the alpha/beta GlcNAc-1-phosphotransferase. Nat. Med..

[bib112] Toyomura A., Fujii T., Kuriki S. (2015). Effect of an 8-week practice of externally triggered speech on basal ganglia activity of stuttering and fluent speakers. Neuroimage.

[bib123] Turk A.Z., Farhan M., Al-Khoury L., Maguire G.A., SheikhBahaei S. (2023). A link between seizure and stuttering disorders? A case report. Annals of clinical psychiatry: official journal of the American Academy of Clinical Psychiatrists.

[bib113] Turk A.Z., Lotfi Marchoubeh M., Fritsch I., Maguire G.A., SheikhBahaei S. (2021). Dopamine, vocalization, and astrocytes. Brain Lang..

[bib114] Van Borsel J., Achten E., Santens P., Lahorte P., Voet T. (2003). fMRI of developmental stuttering: a pilot study. Brain Lang..

[bib115] Watkins K.E., Smith S.M., Davis S., Howell P. (2008). Structural and functional abnormalities of the motor system in developmental stuttering. Brain.

[bib116] Watson B.C., Freeman F.J., Devous M.D., Chapman S.B., Finitzo T., Pool K.D. (1994). Linguistic performance and regional cerebral blood flow in persons who stutter. J. Speech Hear. Res..

[bib129] Wong S.M., Kim J.Y., Maguire G.A. (2021). Migraine and adult-onset stuttering: A proposed autoimmune phenomenon. Annals of clinical psychiatry : official journal of the American Academy of Clinical Psychiatrists.

[bib117] World Health Organization (2015).

[bib118] Wu J.C., Maguire G., Riley G., Fallon J., LaCasse L., Chin S., Klein E., Tang C., Cadwell S., Lottenberg S. (1995). A positron emission tomography [18F]deoxyglucose study of developmental stuttering. Neuroreport.

[bib119] Wu J.C., Maguire G., Riley G., Lee A., Keator D., Tang C., Fallon J., Najafi A. (1997). Increased dopamine activity associated with stuttering. Neuroreport.

[bib120] Yairi E., Ambrose N. (2013). Epidemiology of stuttering: 21st century advances. J. Fluen. Disord..

[bib121] Yairi E. (2007). Subtyping stuttering I: a review. J. Fluen. Disord..

[bib122] Yang Y., Jia F., Siok W.T., Tan L.H. (2017). The role of anxiety in stuttering: evidence from functional connectivity. Neuroscience.

[bib127] Sato Y, Mori K, Koizumi T, Minagawa-Kawai Y, Tanaka A, Ozawa E, Wakaba Y, Mazuka R. Functional lateralization of speech processing in adults and children who stutter. Front Psychol. 2011 Apr 27;2:70. doi: 10.3389/fpsyg.2011.00070. 10.3389/fpsyg.2011.00070 PMC3110423 21687442

