# Supplementary material for: Stuttering as a spectrum disorder: A hypothesis
Source: Curr Res Neurobiol. 2023 Nov 1;5:100116. doi: 10.1016/j.crneur.2023.100116 (PMC10663130; doi:10.1016/j.crneur.2023.100116)
Supplement: Multimedia component 1 [file mmc1.pdf]

Peer Review Overview

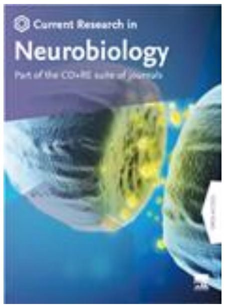

Manuscript Title: Stuttering as a spectrum disorder: a hypothesis

Received	May 27, 2021
1st Decision	Oct 12, 2021
1st Revision Submitted	Nov 01, 2021
2nd Decision	Dec 23, 2021
2nd Revision Submitted	Feb 06, 2022
3rd Decision	Feb 21, 2022
3rd Revision Submitted	Jun 27, 2022
4th Decision	Sep 16,2022
4th Revision Submitted	Sep 26,2023
Accepted	Oct 24, 2023

1st Decision letter

Reference: CRNEUR-D-21-00027
Title: Stuttering as a spectrum disorder: a hypothesis
Journal: Current Research in Neurobiology

Dear Dr. Sheikhbahaei,

Thank you for submitting your manuscript to Current Research in Neurobiology.

I have completed my evaluation of your manuscript. The reviewers and I recommend reconsideration of your manuscript following major revision. I invite you to resubmit your manuscript after addressing the comments below. Please resubmit your revised manuscript by Dec 11, 2021.

When revising your manuscript, please consider all issues mentioned in the reviewers' comments carefully: please outline every change made in response to their comments and provide suitable rebuttals for any comments not addressed. Please note that your revised submission may need to be re-reviewed.

Current Research in Neurobiology values your contribution and I look forward to receiving your revised manuscript.

Kind regards,

Anna S Mitchell, Ph.D.
Editor in Chief
Current Research in Neurobiology

Comments from Editors and Reviewers:

Thank you for your patience with the review process for your manuscript. As you will see the responses of the three reviewers are very different. The journal's editorial team and I have decided to request you complete a major revision of your manuscript, although we cannot guarantee that your paper will be accepted after completing this review. Our evaluation, after considering the reviewers comments, indicates that your manuscript would be of interest to the readers of Current Research in Neurobiology, but it requires substantial changes. We hope that you will carefully consider all of the concerns raised from the reviewers and respond appropriately to them with changes and explanations in your response to the reviewers' comments. After completing this major revision, we will send your paper out for review again.

Reviewer #1:

The authors successfully synthesize current work on genetics and neuroimaging to form the basis for their argument that stuttering is heterogeneous. The call for precision medicine to be applied to stuttering treatment is also applauded. However, I do not think that introducing another model of stuttering is going to advance our field, particularly because the proposed Stuttering Spectrum Disorder model does not make different claims than current multifactorial models, for example, the Multifactorial Dynamic Pathways model (Smith and colleagues). If the authors think that their model is different, they must clearly explain how.

Another criticism is the assumption that stuttering is heterogeneous. There are numerous reasons why stuttering might not be heterogenous. For example, the authors highlight the disparate findings in the neuroimaging literature, which is a problem in stuttering neuro- research. However, there are numerous explanations, for example, that most neuroimaging studies have relatively small Ns (e.g., <30 subjects) and that most studies focus on the fluent speech of speakers who stutter, or when the stutterers are not speaking. In addition, there are consistent differences at a group level, e.g., reduced activation in left- and elevated activation in right speech areas, reduced integrity in left arcuate fasciculus, etc. suggesting perhaps homogeneity. The authors also point to inter-individual variations in phenotype among stutterers as support of their claim of heterogeneity. Indeed, stuttering behaviors manifest themselves differently in different speakers. However, this disparate presentation may have nothing to do with the etiology of stuttering, but rather how individuals respond to the underlying breakdown in speech-language processing that leads to overt stuttering behaviors. An individual's temperament, for example, may shape how their stuttering behaviors are presented. If all speakers had the same temperament, it is possible that all stuttering would manifest itself similarly. It is also worth pointing out that the author's claim that autoimmune disorder causes some stuttering is based on a single case study and a retrospective study. Stuttering may very well be a heterogeneous disorder. However, this cannot be assumed. At the very least, the authors must entertain the possibility that stuttering is not heterogeneous.

Minor concerns:
1. Redundancy - there are several instances of redundancy, for example, the authors discuss autoimmune reactions from lines 218-226, then also include a section entitled, Autoimmune and stuttering."
2. Figure 1 does not, in my view, support the heterogeneity argument or add to the manuscript. It does not seem necessary. All the authors are indicating is that there are subgroups and treatment should be

personalized.
3. There are numerous grammatical errors throughout the manuscript.

Reviewer #2:

This is an outstanding and thought provoking manuscript. Considering stuttering as a spectrum is the best account of the vast variability seen between people who stutter. I particularly appreciated the authors cautions regarding combining data from CWS and AWS for comparison with "control" groups. The overall theme of the manuscript is that stuttering is produced and experienced with a great deal of heterogeneity and the underlying etiologies of stuttering is equally heterogenous. The arguments made and the data presented are well-thought-out and consistent with the clinical experience of working with people who stutter and their families.

Thank you for allowing me the opportunity to review this work.

Reviewer #3:

The goal of the paper is to "propose a new framework for studying and creating personalized treatments for people who stutter." Unfortunately, this paper fails to achieve this commendable goal. It does not provide a comprehensive review of the literature, it does not generate any testable hypotheses, and, in many instances, it misrepresents what we currently know about developmental stuttering, a childhood neurodevelopmental disorder of a multifactorial origin. The proposed Stuttering Spectrum Disorder (SSD) model does not meet basic requirements of a model: it does not explain or predict stuttering behaviors, it is not supported by scientific data (as currently written) and, as I have already mentioned, it does not generate any testable hypotheses.

The authors state that the Stuttering Spectrum Disorder (SSD) model recognizes the existence of heterogeneity among people who stutter, which I think is already a well-established and accepted notion in the field of stuttering research and clinical practice.
The authors put the term "childhood onset fluency disorder" in the keywords but the scientific literature discussing stuttering in children who stutter is largely absent from their review. As stuttering first emerges in early childhood (2-4 years of age), the discussion of stuttering phenotype in adults only appears problematic. Many of stuttering characteristics in adults are the result of life-long stuttering (and its adverse impact) and not the cause(s) of the disorder (as they are generally not observed in young children who stutter).
The authors rely heavily on the APA Diagnostic and Statistical Manual of Mental Disorders when describing the characteristics of stuttering, which is problematic in my opinion. For a theoretical model, such as this, the authors should cite original sources (peer-reviewed research articles) to support their statements.
My other, section specific, comments are included below.
Lines 51-53: What "current model" are the authors referring to? It is well-established that stuttering is a neurodevelopmental disorder of a multifactorial origin and that it has diverse phenotypes/characteristics.
Line 53: Do we know of genetic factors that determine stuttering subtypes? If so, they should be included in the paper.
Line 56-59: Could the authors be specific about how they group people who stutter based on their genetics and phenotypes. This information currently is not included in the manuscript.

Line 59: Because the model (in its current form) lacks any specific details, it cannot facilitate development of personalized treatments for people who stutter. Little if any data are presented that could inform treatment decisions for people who stutter.
Lines 70-71: The authors must be specific which "speech disorders" they refer to. There are other speech disorders besides stuttering.
Lines 71-72: The statement that "the majority of children who stutter (CWS) recover by their adolescence" is misleading as the majority of CWS who "outgrow" stuttering do so in their preschool years.
Lines 74-80: I think it is misleading to say that "no treatment has yet been approved by FDA to address the disfluency of speech". What kinds of treatments does FDA regulate? Current evidence-based treatments for stuttering are behavioral, would they be regulated by the FDA? Do the authors imply that no treatment other than a drug treatment could help people who stutter?
Lines 83-85 Please include most current published articles on stuttering genetics (some of the more recent references appear later in the manuscript). Do we have a consensus on what genetic mutations cause stuttering? I think including this information in support of SSD would be helpful.
Lines 86-87: I am very interested in learning more about the proposed basal ganglia involvement in stuttering. If I remember correctly, Wu et al papers (1995, 1997) were based on observations of three adults who stutter, and their findings have not been replicated. I think this level of evidence is not enough to support a model. Similarly, Alm 2020 paper is a "hypothesis paper". Given that, I don't think it can support a model either.
Lines 107-113: Please cite original peer-reviewed sources rather than the APA DSM5 when describing stuttering behaviors. Please include the information on stuttering characteristics of young children who stutter (this body of literature is all but missing from the manuscript).
Lines 112-113: Clarification is needed here: do the authors propose that perspiration and cardiorespiratory events are unique to stuttering or are a natural reaction to the communication difficulties and negative listener feedback? In similar vein, anxiety and avoidance are typically learned reactions that come about because of communication difficulties (these are not typically seen in young children who stutter). I see that the authors acknowledge the latter, but I am not sure how it supports the SSD model.
Lines 129-155: I appreciate the review of the imaging literature, but would like to see studies with children who stutter represented as well.
Line 154: Please double check your references. Walsh et al 2015 examined behavioral manifestations of stuttering and does not offer any data to support the "functional connectivity of the basal ganglia thalamocortical loop".
Lines 157-161: Based on my reading on the literature, rarely if any study published in the last 15 years combined data from children and adults who stutter and treated them as a homogeneous group. If there are studies like that, I think the authors could include specific references. In the paragraph above, however, this is what the authors are seemingly doing in their discussion of differences in brain anatomy in people who stutter.
Lines 171-175: Please include a more balanced review of research on treatments for stuttering. Are the authors suggesting that children be treated with dopamine-blocking medications rather than behavioral treatments? In line 173, it is implied that behavioral therapies "were associated with greater relapse rates", greater than what other treatment? Please clarify.
I think expanding the discussion of genetic research on stuttering (lines 201-257) and connecting this work to different stuttering phenotypes would support the SSD model, this however is not done in the current version of the manuscript.
Similarly, the section on dopamine and stuttering and autoimmune "response" (please check the heading) and stuttering (lines 283-296) is very interesting but rather vague. I hope to see more

connections to the proposed stuttering subtypes. Also, please include more details on "current promising clinical trials" (lines 213-216). It would also be helpful to know how prevalent is stuttering as a result of bacterial infections. Alm's paper, if I remember correctly, was discussing historical accounts from the time before the advent of antibiotics.
Thank you for the opportunity to review the above referenced manuscript. I hope my suggestions are helpful as the authors further develop their model.

1st Author Response Letter

Response to comments from Editors and Reviewers:

We would like to thank all three reviewers and the Editors of Current Research in Neurobiology for their time taken to evaluate our submission. We are grateful for the detailed and mostly constructive comments provided and delighted to have an opportunity to re-submit our perspective. We now include additional information requested by the reviewers, provide a full response to all the criticisms raised and submit a thoroughly revised manuscript. We believe very strongly that the revised manuscript fully addresses all the criticisms raised and further support the hypothesis that stuttering could be considered as a spectrum disorder. Similar to the diverse comments that we have received from the esteemed reviewers, we deem this hypothesis will stimulate great discussions in the field as well. Below we state the criticisms and then provide our responses.

Comments from Reviewer 1:

The authors successfully synthesize current work on genetics and neuroimaging to form the basis for their argument that stuttering is heterogeneous. The call for precision medicine to be applied to stuttering treatment is also applauded. However, I do not think that introducing another model of stuttering is going to advance our field, particularly because the proposed Stuttering Spectrum Disorder model does not make different claims than current multifactorial models, for example, the Multifactorial Dynamic Pathways model (Smith and colleagues). If the authors think that their model is different, they must clearly explain how.

Response: We would like to thank this reviewer for his/her time taken to review our manuscript. We agree with the reviewer that the current work on genetics and neuroimaging strongly suggests that stuttering is heterogeneous disorder. However, we respectfully disagree with the reviewer and strongly believe that a new framework is much needed considering the current discoveries. The multifactorial dynamic pathways theory of stuttering emphasizes the dynamic developmental context in which stuttering emerges and follows its progression throughout early childhood. But, as we know the majority of children who stutter recover by their adolescence, though the mechanism(s) are not known. In addition, these models do not incorporate components of pharmacoor gene-therapies. Recent data indicate that people who stutter who have mutations in lysosomal targeting pathways do not respond well to speech therapy (FrigerioDomingues et al., 2019). What would the multifactorial dynamic pathways theory provide for this sub-group of people who stutter? What about the people who stutter who have other genetic mutations? Therefore, in the authors’ opinion there is a critical need for a more comprehensive framework in which adults who stutter be provided guidance of personalized medicine in their respective, optimal form of therapy. We have described these specific details in lines 306 - 331.

Another criticism is the assumption that stuttering is heterogeneous. There are numerous reasons why stuttering might not be heterogenous. For example, the authors highlight the disparate findings in the neuroimaging literature, which is a problem in stuttering neuro- research. However, there are numerous explanations, for example, that most neuroimaging studies have relatively small Ns (e.g., < <30 subjects) and that most studies focus on the fluent speech of speakers who stutter, or when the stutterers are not speaking.

Response: We respectfully disagree with this comment. A significant barrier to effective research, diagnosis, and treatment of stuttering has been the practice of investigators and clinicians to view it as a single disorder in spite of its immense diversity.

Similarly, we do not believe that published, peer reviewed neuroimaging data in the field of stuttering is problematic. The data strongly suggest that there is heterogeneity among stuttering subjects. An alternate explanation for 'disparate findings in the neuroimaging literature' points toward the nature of human speech, which is the most complex motor behavior which requires coordination of activities of more than 100 muscles in the body and many circuits in the brain. One can imagine that any defect in any of these brain circuits would lead to the stuttering phenotype. We do not believe that one treatment plan will work on all the stuttering cases just as not one therapy works on all forms of other basal ganglia-related conditions such as OCD or Tourette Syndrome.

On the other hand, as stated in the original submission (line 159-161) we believe the conventional methodologies of imaging studies are problematic because researchers divide people based on their phenotype (in this case having or not having stuttering phenotypes) into experimental groups. Therefore, these imaging studies may not be appropriate and may mask important findings.

In our opinion, n = 30 for an imaging study is not small. We must ask what is an acceptable N for this reviewer? Where the line should be taken in terms of the acceptable N? and most importantly, why having a higher sample size in imaging studies would lead to less heterogeneous results? We proposed in this perspective, that imaging data should be combined by genetics and phenotype analysis and only people with similar traits be placed in the experimental group.

In addition, several studies conducted their imaging studies during the act of stuttering (for instance, see Wu et al, Fox et al, and Braun et al studies). Moreover, as opposed to the reviewer's opinion, we strongly believe that resting state (RS) imaging is very important and could be informational. The principle of RS-imaging is based on the same principle as active-task imaging. These studies can be done in adults as well as pediatric subjects to study the normal structure/connectivity of the brains.

In addition, there are consistent differences at a group level, e.g., reduced activation in left- and elevated activation in right speech areas, reduced integrity in left arcuate fasciculus, etc. suggesting perhaps homogeneity.

Response: We are not sure if we understand the reviewer's comment. If the reviewer is suggesting that the imaging studies uniformly show "reduced activation in left- and elevated activation in right speech areas, reduced integrity in left arcuate fasciculus, etc." in all the "stuttering groups" (i.e., all the stuttering group in all the studies) then we invite the reviewer to read the entirety of the studies cited in this section (lines 130-163).

The authors also point to inter-individual variations in phenotype among stutterers as support of their claim of heterogeneity. Indeed, stuttering behaviors manifest themselves differently in different

speakers. However, this disparate presentation may have nothing to do with the etiology of stuttering, but rather how individuals respond to the underlying breakdown in speech-language processing that leads to overt stuttering behaviors. An individual's temperament, for example, may shape how their stuttering behaviors are presented. If all speakers had the same temperament, it is possible that all stuttering would manifest itself similarly.

Response: We respectfully disagree with the reviewer. In our opinion, the fact that there are "individuals respond to the underlying breakdown in speech-language processing" strongly suggest the disorder is heterogeneous. To follow reviewer's logic, not all people who stutter possess the same temperament. Therefore, the manifestation of stuttering differs among people. We and others hypothesized that this difference is due to the heterogeneity of the disorder, based on phenotypes, imaging data and varied responses to treatment.

Moreover, in this perspective, we have focused on the known etiologies of stuttering. These etiologies are significantly different. We acknowledge that there are other etiologies that will be discovered in the future and each one may require a different therapy.

It is also worth pointing out that the author's claim that autoimmune disorder causes some stuttering is based on a single case study and a retrospective study.

Response: There are three case reports of stuttering associated with GAS infections (PMCID: PMC7693426). The 'retrospective study' by Alm (2020) strongly correlate the stuttering disorder with GAS infections. In our opinion, it is plausible that this mechanism can still lead to stuttering. The autoimmune response does not necessarily have to be related to the GAS infection though. In our opinion, any immune reactions against dopamine receptors of the basal ganglia during a critical period of language development can lead to disorder in speech motor control, as it has been shown to cause other movement disorders (See PMID: 16225742; PMCID: PMC3398718; and others).

However, to respond to this comment of the reviewer, we have updated the text of the revised manuscript.

"it is possible that eliminating the underlying infection or decreasing the immune system reactivities might be helpful and no other interventions might be required. Additional research, possibly in animal models, are required to further expand the details of this possible stuttering etiology."

Stuttering may very well be a heterogeneous disorder. However, this cannot be assumed. At the very least, the authors must entertain the possibility that stuttering is not heterogeneous.

Response: We respectfully disagree. This perspective was submitted as a "Hypotheses & Intersections" article type to the Current Research in Neurobiology journal. We believe we have provided strong evidence that stuttering is a heterogeneous disorder. Both reviewers 2 and 3 believe that the notion of heterogeneity in stuttering "is already a well established and accepted notion in the field of stuttering research and clinical practice."

Minor concerns:

1.Redundancy - there are several instances of redundancy, for example, the authors discuss autoimmune reactions from lines 218-226, then also include a section entitled, Autoimmune and

stuttering."

Response: We thank the reviewer for this comment. These redundancies were intentional. First, we described the known etiologies of stuttering briefly in lines 218-226, and then we dived deeper in each of the and gave them a separate section.

2. Figure 1 does not, in my view, support the heterogeneity argument or add to the manuscript. It does not seem necessary. All the authors are indicating is that there are subgroups and treatment should be personalized.

Response: We thank the reviewer for this comment. The message of this perspective is that "there are subgroups and treatment should be personalized." Therefore, we are happy that the figure conveys this message. In Figure 1, the color coding of the people and the ratio of female to male (1:4) all supports the heterogeneity argument. However, to address this comment of the reviewer, we have now added this important information to the figure legend.

3. There are numerous grammatical errors throughout the manuscript.

Response: we thank the reviewer for this comment. The revised manuscript is edited extensively. Thank you.

Comments from Reviewer 2:

This is an outstanding and thought provoking manuscript. Considering stuttering as a spectrum is the best account of the vast variability seen between people who stutter. I particularly appreciated the authors cautions regarding combining data from CWS and AWS for comparison with "control" groups. The overall theme of the manuscript is that stuttering is produced and experienced with a great deal of heterogeneity and the underlying etiologies of stuttering is equally heterogenous. The arguments made and the data presented are well-thought-out and consistent with the clinical experience of working with people who stutter and their families. Thank you for allowing me the opportunity to review this work.

Response: We would like to thank this referee for her/his time taken to review our perspective and very positive assessment of our work. We agree that considering "stuttering as a spectrum is the best account of the vast variability seen between people who stutter."

Comments from Reviewer 3:

The goal of the paper is to "propose a new framework for studying and creating personalized treatments for people who stutter." Unfortunately, this paper fails to achieve this commendable goal. It does not provide a comprehensive review of the literature, it does not generate any testable hypotheses,

Response: We would like to thank this reviewer for his/her time taken to review our manuscript. We respectfully disagree with the reviewer and strongly believe that the manuscript provides a compelling argument for a 'hypothesis' that stuttering can be considered as spectrum disorder; our proposed framework is our hypothesis, as we have submitted the manuscript as a "Hypotheses & Intersections" article type! We have now clearly stating this in the title of our manuscript as well.

In addition, this manuscript was not a review on 'stuttering disorder,' rather a perspective (Line 91

original submission). Therefore, we believe that expecting a ‘comprehensive review of the literature,’ is vague and unreasonable expectation. However, to further clarify the manuscript, we have added the type (i.e., perspective) to the abstract, introduction, and conclusion.

This reviewer has made several assumptions regarding this manuscript that are not entirely accurate. First, our submission is a review article; it is not and our manuscript is a perspective article. Second, there is no hypothesis; the submission is our hypothesis, which is stuttering can be considered as a spectrum disorder. Third, the SSD is a model to diagnose stuttering at a younger age. SSD refers to a broad range of conditions characterized by stuttering phenotype. We have now added this information to the perspective. (Lines 330-331)

and, in many instances, it misrepresents what we currently know about developmental stuttering, a childhood neurodevelopmental disorder of a multifactorial origin.

Response: We believe that this comment may represent this reviewer’s alternative view on the pathophysiology of stuttering. Respectfully, from the comments provided we were unable to envisage the reviewer’s perspective of which ‘currently known facts’ are misrepresented. All data presented in this ‘perspective’ are based on the published, peer reviewed literature.

The proposed Stuttering Spectrum Disorder (SSD) model does not meet basic requirements of a model: it does not explain or predict stuttering behaviors, it is not supported by scientific data (as currently written) and, as I have already mentioned, it does not generate any testable hypotheses.

Response: As mentioned above, our ‘perspective’ was not designed to be exhaustive review. Also, we do not know what the reviewer means by ‘predicting stuttering behavior.’ In our view, the current ‘scientific data’ suggests that “stuttering is produced and experienced with a great deal of heterogeneity and the underlying etiologies of stuttering is equally heterogenous,” (as clearly stated by reviewer 2). As mentioned above, this perspective is submitted as a “Hypotheses & Intersections” article type. The proposed framework is our hypothesis. However, to clarify this issue, we removed any references to ‘model’ from the revised manuscript and added hypothesis to the tile. In addition to this general comment, some references pointing to this comment would have been helpful.

The authors state that the Stuttering Spectrum Disorder (SSD) model recognizes the existence of heterogeneity among people who stutter, which I think is already a wellestablished and accepted notion in the field of stuttering research and clinical practice.

Response: We respectfully disagree with the reviewer and invite the reviewer to read the comments from reviewer number 1. In this instance, 1 out of 3 people does not believe in the ‘well-established and accepted notion’ that stuttering is a heterogeneous disorder! Therefore, we believe the discussion about heterogeneity of the stuttering under the SSD framework is required.

The authors put the term "childhood onset fluency disorder" in the keywords but the scientific literature discussing stuttering in children who stutter is largely absent from their review. As stuttering first emerges in early childhood (2-4 years of age), the discussion of stuttering phenotype in adults only appears problematic. Many of stuttering characteristics in adults are the result of life-long stuttering (and its adverse impact) and not the cause(s) of the disorder (as they are generally not observed in young children who stutter).

Response: We respectfully point to the fact that this ‘perspective’ is not a review. In addition, childhood-onset fluency disorder, in our humble opinion, means that the fluency disorder (i.e., stuttering in this case) is started from the childhood and if we are studying it in adults, it should have been persisted. In our opinion, up to now (Oct 15, 2021), there are only few solid causes of stuttering is presented in the literature and we have extensively discussed those. We agree with the reviewer that “Many of stuttering characteristics in adults are not causes of disorder.” However, this perspective is not about stuttering characteristics. We agree that a new framework to describe

stuttering in children who stutter might be necessary, but it is outside the scope of our current perspective. We have now clearly stated that in the revised manuscript. "Since about 90% of CWS recover spontaneously with or without intervention (Yairi and Ambrose, 2013), in this perspective, we have mainly focused on adults who stutter (AWS) and based on inter-individual variations of stuttering phenotypes, imaging studies, and responses to treatments/therapies further argue for heterogeneity of stuttering disorders. In this context, we propose a new frame-work for studying and creating personalized treatment plans for AWS."

The authors rely heavily on the APA Diagnostic and Statistical Manual of Mental Disorders when describing the characteristics of stuttering, which is problematic in my opinion. For a theoretical model, such as this, the authors should cite original sources (peer-reviewed research articles) to support their statements.

Response: We agree with the reviewer and in the revised manuscript, we have cited more original studies. However, in this perspective, we do not talk about stuttering phenotype per se. We argued that the phenotypes of stuttering are diverse and this diversity points toward the heterogeneity of stuttering disorders. For diagnosis of stuttering disorders, symptoms should follow the APA Diagnostic and Statistical Manual of Mental Disorders guideline.

Lines 51-53: What "current model" are the authors referring to? It is well-established that stuttering is a neurodevelopmental disorder of a multifactorial origin and that it has diverse phenotypes/characteristics.

Response: We thank the reviewer for this comment. We agree with the reviewer that this sentence is not as clear as we expected it to be, and we have revised the sentence now.

Line 53: Do we know of genetic factors that determine stuttering subtypes? If so, they should be included in the paper.

Response: In our opinion, works done by Dr. Drayna and colleagues in discovering the genes that associated with stuttering in explored in detail in the manuscript (lines 234- 263). However, we proposed that these mutations only exist in a subgroup of people who stutter, and other mutations are likely to be involved in pathophysiology of stuttering. Studies from Dr. Shelly Jo Kraft will shed some light in this issue.

Line 56-59: Could the authors be specific about how they group people who stutter based on their genetics and phenotypes. This information currently is not included in the manuscript.

Response: In this perspective we proposed just placing a group of people who stutter in one experimental group and performing an imaging or therapeutic study is not sufficient anymore. In addition, we have clearly provided a road map for future studies under the SSD framework (lines 312-323 in original submission)

Line 59: Because the model (in its current form) lacks any specific details, it cannot facilitate development of personalized treatments for people who stutter. Little if any data are presented that could inform treatment decisions for people who stutter.

Response: We respectfully disagree. We have submitted the manuscript as a "Hypotheses & Intersections" article type. Our proposed framework is our hypothesis and we have proposed specific treatment for discovered subtypes of stuttering (genetic causes, dopamine hypothesis, and autoimmune reactions), which are discussed in detailed in the manuscript (lines 228-296 original submission).

Lines 70-71: The authors must be specific which "speech disorders" they refer to. There are other speech disorders besides stuttering.

Response: We are totally surprised by this comment! As it is stated and can be deduced from the text,

we mean all types of the speech disorders.

Lines 71-72: The statement that "the majority of children who stutter (CWS) recover by their adolescence" is misleading as the majority of CWS who "outgrow" stuttering do so in their preschool years.

Response: We believe that this comment may represent this reviewer's alternative view on the pathophysiology of stuttering. We also do not know the scientific definition of 'outgrew.' In addition, the reviewer mentioned above that "As stuttering first emerges in early childhood (2-4 years of age)," and now claiming that it goes away in the preschool years. We find these comments contradictory, and at the very least some reference would be helpful to understand the reviewer's point of view. In addition, the concept of recovery from stuttering is complex and estimations of recovery rate are likely to be greatly affected by differences in definitions and measurement across studies (see PMID: 32445989). Therefore, we believe that it is safe to say that most of the CWS recover by adolescence.

Lines 74-80: I think it is misleading to say that "no treatment has yet been approved by FDA to address the disfluency of speech". What kinds of treatments does FDA regulate? Current evidence-based treatments for stuttering are behavioral, would they be regulated by the FDA? Do the authors imply that no treatment other than a drug treatment could help people who stutter?

Response: We respectfully disagree with the reviewer and stand by our claim that FDA has not approved ANY treatment for stuttering yet. A range of treatments, based on the published & peer reviewed papers, is proposed in this perspective for each sub-type of stuttering (lines 228-296 in the original manuscript). In addition, we have clearly stated in the manuscript that"…speech and cognitive therapies remain first-line oftreatment." Therefore, we strongly believe the reviewer comment that "Do the authors imply that no treatment other than a drug treatment could help people who stutter?" is misleading and may represent this reviewer's alternative view.

Lines 83-85 Please include most current published articles on stuttering genetics (some of the more recent references appear later in the manuscript). Do we have a consensus on what genetic mutations cause stuttering? I think including this information in support of SSD would be helpful.

Response: The message of our perspective is that there is no one genetic mutation link to all types of stuttering disorders. In our opinion, up to now (October 15th, 2021) the most solid work on genetic mutations that are linked to stuttering is provided by Dr. Drayna and colleagues and has been extensively discussed in the original manuscript (lines 202- 210 & 228-257). However, to address the comment of the reviewer, we have revised the text and added the following statement. "For instance, mutations in gene ARNT2 is also linked to stuttering (Kraft, 2010), however, more experiments are needed to clarify the function of this gene in pathophysiology of stuttering [for a recent update on the possible role of this gene, see (Alm, 2021)]" (lines 212-215)

Lines 86-87: I am very interested in learning more about the proposed basal ganglia involvement in stuttering. If I remember correctly, Wu et al papers (1995, 1997) were based on observations of three adults who stutter, and their findings have not been replicated. I think this level of evidence is not enough to support a model. Similarly, Alm 2020 paper is a "hypothesis paper". Given that, I don't think it can support a model either.

Response: Again, we believe that this comment may represent this reviewer's alternative view on the pathophysiology of stuttering. Wu et al studies were based on PET imaging in four adults who stutter (cited by 257 papers up to now). The statement that Wu et al's findings were not replicated is misleading; it is our knowledge that no one has yet attempted to replicate studies of dopamine imaging or FDG PET in stuttering. On the other hand, data from Watkins et al (see also a preprint manuscript by Watkins and colleagues https://osf.io/ypq8s) and Chang et al (see their latest review in 2020) suggesting the role of the striatum in pathophysiology of stuttering. Works of Alm (2004) and Busan et al (2005) supporting the central role of dopamine in stuttering. Wu et al finding was further

evaluated and expanded in Alm 2004 (PMID: 15159193), and validated by a computational model by Civier and colleagues (PMID: 23872286). This hypothesis is also supported by genetics studies (PMID: 19590515), and most importantly by the current paper that shows accumulation of iron in basal ganglia (and other brain regions involve in vocal production) (PMID: 33643015; see also a preprint manuscript by Watkins and colleagues https://osf.io/ypq8s). In our view, all these data, point toward involvement of basal ganglia in a subgroup of people who stutter. In addition, basal ganglia are involved in basal ganglia-thalamo-cortical loop that has been proposed to have a critical role in speech production in humans (see PMID: 32047456). In addition, 3 case reports suggest that autoimmune reactions (against dopamine receptors in the basal ganglia) can be related to the stuttering phenotype. These data are now expanded and explored in Alm 2020. In our opinion, these scientific data and model is sufficient to propose that autoimmune reactions can be related to stuttering phenotype and CAN be considered as a sub-type of stuttering disorders. However, to answer to this comment of the reviewer, we have revised the text of the manuscript and added, "Additional research, possibly in animal models, are required to further expand the details of this possible stuttering etiology."

Lines 107-113: Please cite original peer-reviewed sources rather than the APA DSM5 when describing stuttering behaviors. Please include the information on stuttering characteristics of young children who stutter (this body of literature is all but missing from the manuscript).

Response: We thank the reviewer for this comment. We have now cited some of original studies as well. However, as we mentioned before, behavioral characteristics of young children who stutter is out of the scope of this perspective, as in this perspective we have focused on the AWS. We have explicitly stated this in the revised manuscript.

Lines 112-113: Clarification is needed here: do the authors propose that perspiration and cardiorespiratory events are unique to stuttering or are a natural reaction to the communication difficulties and negative listener feedback? In similar vein, anxiety and avoidance are typically learned reactions that come about because of communication difficulties (these are not typically seen in young children who stutter). I see that the authors acknowledge the latter, but I am not sure how it supports the SSD model.

Response: In lines 112-113, we are just repeating existing evidence on the heterogeneity of stuttering phenotypes in people who stutter and simply stating that in some people who stutter, stuttering is associated with perspiration and cardiorespiratory events. We believe it is clearly stated that these autonomic events "could be present concurrently" (Line 113 original submission). We do not propose that these events are unique to stuttering nor are a natural reaction to stuttering. Regardless of their origin, not all the people who stutter, show these autonomic events. It seems that reviewer's comments on anxiety and avoidance is related to lines 115-121. They are clearly presented under the sub-section "Inter-individual variations of stuttering phenotype." So logically, by presenting information/data, we argue that the stuttering phenotype is heterogeneous among the PWS. In addition, several lines of research, strongly suggested that anxiety and avoidance behaviors are in fact innate behaviors and the brain circuitry for these behaviors are defined as well (for instance, see these recent papers published in high profile journals: PMCID: PMC4780260; PMCID: PMC4780260; PMCID: PMC8221601; PMCID: PMC4035371). Moreover, the extreme case of generalized anxiety disorder (GAD) offers perhaps the most compelling case for the relevance of innate anxiety. However, to address the reviewer's comment, we have now updated the text to convey the heterogeneity of the symptoms more clearly.

Lines 129-155: I appreciate the review of the imaging literature, but would like to see studies with children who stutter represented as well.

Response: We have focused on childhood-onset stuttering disorder in adults who stutter and

discussing imaging studies of children who stutter is outside the scope of this perspective. In the revised text, we have clearly stated that (Lines 88-93).

Line 154: Please double check your references. Walsh et al 2015 examined behavioral manifestations of stuttering and does not offer any data to support the "functional connectivity of the basal ganglia thalamocortical loop".

Response: We thank the reviewer for the comment and apologize for this mistake. In the revised version of the manuscript, we have updated the references.

Lines 157-161: Based on my reading on the literature, rarely if any study published in the last 15 years combined data from children and adults who stutter and treated them as a homogeneous group. If there are studies like that, I think the authors could include specific references. In the paragraph above, however, this is what the authors are seemingly doing in their discussion of differences in brain anatomy in people who stutter.

Response: We do not understand this comment of the reviewer. This section of the manuscript was specifically applauded by reviewer 2. Therefore, we believe this comment may represent reviewer's point of view. If the reviewer imply that we mean researchers combine data gathered from CWS and AWS, this was not what we indented to convey. To further clarify our point, we have changed the text to "AWS." If the reviewer suggesting that in imaging data, researchers do not have a group called AWS (or CWS) and the characteristics of this group is that all the member of this group stutter, then we recommend the reviewer to re-read the last 15 years of literature! This is exactly our concern with the imaging data. In this perspective, we propose that researchers should consider the genetics and characteristics of the stuttering phenotype when performing imaging data and just grouping AWS or CWS based on the fact that they show stuttering phenotype may mask important information.

Lines 171-175: Please include a more balanced review of research on treatments for stuttering. Are the authors suggesting that children be treated with dopamine-blocking medications rather than behavioral treatments? In line 173, it is implied that behavioral therapies "were associated with greater relapse rates", greater than what other treatment? Please clarify.

Response: In this perspective, we have clearly stated that, "…no therapeutic agent has yet been approved by the Food and Drug Administration (FDA) for the treatment of stuttering, and speech and cognitive therapies remain first-line treatment." We have also discussed different speech and behavioral therapies [such as direct and indirect Lidcombe program, cognitive behavioral therapy (CBT), as well as transcranial direct current stimulation (tDCS), repetitive transcranial magnetic stimulation (rTMS), and possible pharmacology therapies. In our view, we have provided a balanced view on available treatment for stuttering. It would have been helpful to know the viewpoint of the reviewer on where she/he believes there is an imbalance in our discussion. Respectfully we do not fully understand the relevance of the reviewer's question on prescribing dopamine-blocking medication to children, and again, we believe that this comment may represent this reviewer's alternative view on the pathophysiology of stuttering. We have now clearly stated that this perspective is focused on AWS (Lines 88-93). We thank the reviewer's comment for line 173 and we apologize for the confusion. We have fixed the text now.

I think expanding the discussion of genetic research on stuttering (lines 201-257) and connecting this work to different stuttering phenotypes would support the SSD model, this however is not done in the current version of the manuscript.

Response: We do not understand what the reviewer means by "connecting genetic research on stuttering to different stuttering phenotypes?" Respectfully, genes and mutations in genes do not cause stuttering on their own, rather these mutations have to modify brain circuits that are involved in complex motor behavior of speech production. The most solid work on genetics of stuttering is

done by Dr. Drayna and colleagues; they have identified four genes related to the lysosomal enzyme-targeting pathway that are linked to stuttering behaviors in humans and mouse models. However, the mechanism by which these mutations (and other mutations) might lead to stuttering is not known at the moment. Several groups are working on this problem. For instance, recently it was published that a mutation in these genes is associated with altered gray matter volume in children with persistent stuttering (PMID: 34041495). Again, it is yet to be defined that why alteration of gray matter might lead to stuttering disorder.

Similarly, the section on dopamine and stuttering and autoimmune "response" (please check the heading) and stuttering (lines 283-296) is very interesting but rather vague. I hope to see more connections to the proposed stuttering subtypes.

Response: We do not understand the comment of the reviewer either. In the perspective we argue that since known etiologies of stuttering are broad, the treatment should be broad. We propose that stuttering can be studies under SSD framework, in which the SSD refers to a broad range of conditions characterized by stuttering phenotype. We have discussed them in detail in lines 306-322 of the original manuscript.

Also, please include more details on "current promising clinical trials" (lines 213-216).

Response: The requested details were provided in lines 190-199 of the original submission. Since this perspective is not about treatment of stuttering disorders with pharmacological agents, to address this comment of the reviewer, we added, “see (Maguire et al., 2020) for a comprehensive review on this topic” (lines 192-193)

It would also be helpful to know how prevalent is stuttering as a result of bacterial infections. Alm's paper, if I remember correctly, was discussing historical accounts from the time before the advent of antibiotics.

Response: We believe the author refers to lines 218-226 of the original manuscript. Again, we believe that this comment may represent this reviewer’s alternative view on the pathophysiology of stuttering. There is no data on linking ‘bacterial infection’ to stuttering! Rather there are clinical data and now a model (Alm 2020) for how stuttering might be associated with a specific group of streptococcal infections that can cause autoimmune reactions (against dopamine receptors in the basal ganglia). the authors strongly believe that, since it happened before, untreated (specific) streptococcal infections still might lead to stuttering disorders.

Thank you for the opportunity to review the above referenced manuscript. I hope my suggestions are helpful as the authors further develop their model.

2nd Decision letter

Reference: CRNEUR-D-21-00027
Title: Stuttering as a spectrum disorder: a hypothesis
Journal: Current Research in Neurobiology

Dear Dr. Sheikhbahaei,

Thank you for submitting your manuscript to Current Research in Neurobiology.

I have completed my evaluation of your manuscript. The reviewers recommend reconsideration of your manuscript following major revision. I invite you to resubmit your manuscript after addressing the comments below. Please resubmit your revised manuscript by Feb 21, 2022.

When revising your manuscript, please consider all issues mentioned in the reviewers' comments carefully: please outline every change made in response to their comments and provide suitable rebuttals for any comments not addressed. Please note that your revised submission may need to be re-reviewed.

Current Research in Neurobiology values your contribution and I look forward to receiving your revised manuscript.

Kind regards,

Anna S Mitchell, Ph.D.
Editor in Chief
Current Research in Neurobiology

Comments from Editors and Reviewers:

Please can you specifically address both of the reviewers comments in your next revision and explain how you've address these comments in your response to reviewers.

Reviewer #1:

The authors have addressed my comments, except for their continued claim that stuttering IS heterogenous. It may be--this is their hypothesis. I think the authors provide some support for their argument. However, stuttering might not be heterogenous. For example, the "heterogeneity in results of both anatomical and functional human imaging studies," may reflect heterogeneity in the disorder, but may also reflect spurious findings due to small sample sizes, differing tasks, etc. To answer the authors' question, 30 is probably at the low end of a sufficient sample size, statistically. 30 is the extreme upper end of the range for stuttering studies (e.g., Fox et al., 1996 -> 10 AWS; Ingham et al., 2003 -> 10 AWS; Watkins et al., 2008 -> 12 AWS; Sommer et al., 2002 -> 15 AWS; Chang et al., 2009 -> 20 AWS). In an age of data dredging, it would be productive for researchers of stuttering to look at consistent findings vs. assuming all results are "right." My concern is that the authors are pitching testable hypotheses as facts. Thus, I think at the very least, the authors must soften their language to reflect that stuttering MAY be heterogenous in origin. The authors do this in some places, but not others, e.g.,
1) "As discussed above, known etiologies of stuttering (including genetics, excess dopamine, autoimmune response, and maybe others) are varied." The etiology is unknown.
2) "Stuttering is a heterogeneous disorder as etiology, phenotype, diagnostics, and responses to treatments differ among people with stuttering." Stuttering MAY be heterogenous.

Reviewer #3:

I have carefully read the authors' response and the revised manuscript. The revised manuscript has minimal stylistic changes, and most comments and suggestions from Reviewer 1 and me have been dismissed. In their response to Reviewer 1 and me the authors keep referring to some "alternative view" on the pathophysiology of stuttering that I and the other reviewer seemingly hold. I do not know what the authors mean by the "alternative view"; using this argument to dismiss comments from your reviewers seems unproductive. I still stand by my comments and invite the authors to revise their

manuscript and present a more convincing argument supported by a thorough and comprehensive literature review (including data from children who stutter). Stuttering is a childhood disorder, children who stutter grow up to be adults who stutter (and develop learned behaviors in the process). Discussion of the etiology of stuttering based exclusively on adult data greatly limits the usability of the authors' perspective. Yet, the authors do not mention any limitations or caveats of their perspective.
The authors state (lines 51-56) that "The Stuttering Spectrum Disorder (SSD) framework also improves the conventional model by grouping people who stutter based on their genetics and manifestation of stuttering, and therefore provides targeted treatment to the subgroups of adults/children who stutter. The SSD framework may facilitate recent efforts of precision medicine to provide personalized treatment plans to people with stuttering."
I would like to see a detailed explanation supported by research references for each of the above claims: please explain how people who stutter should be grouped based on their genetics and manifestation of stuttering, please explain what treatments should be applied to what subgroups of people who stutter. It seems that the proposed stuttering heterogeneity mentioned in the manuscript has to do with the possible causes of stuttering. It could be related to four or five genetic mutations (what differences in brain circuits do these mutations cause?). It could be related to a basal ganglia dysfunction (what genes are involved in this etiology?). It could also be an autoimmune reaction to a streptococcal infection, which seemingly also leads to a basal ganglia dysfunction. Do these mechanisms represent three unique etiologies or are they related? How is stuttering phenotype or "manifestation of stuttering" similar or different in people with the three proposed etiologies? Please provide a detailed discussion in the manuscript.
The authors state that their perspective "improves the conventional model by grouping people who stutter based on their genetics and manifestation of stuttering". Which genetic mutations constitute a basis for a stuttering subgroup? If I were to plan a research study today, what criteria would I use to divide people who stutter into subgroups? In their response to my prior comment about this issue, the authors referred me to this paragraph:
"With improved imaging techniques, next-generation DNA sequencing technologies, advanced electrophysiology, availability of animal models, and sophisticated deep learning algorithms, more studies should be done to define subtypes of the stuttering disorders under the umbrella of stuttering spectrum disorders (SSD). Combining genomic testing with next-generation analyses of imaging data will help clinicians to set criteria for each SSD sub-type and predict if a person with SSD would respond to certain therapies."
Respectfully, the paragraph above provides little if any information on how to classify people who stutter into subgroups or define stuttering subtypes.

In closing, the SSD perspective is currently poorly defined and presents researchers with an impossible task of grouping people who stutter based on unspecified characteristics.

2nd Author Response Letter

Response to comments from Editors and Reviewers:

Comments from Reviewer 1:

The authors have addressed my comments, except for their continued claim that stuttering IS heterogenous. It may be--this is their hypothesis. I think the authors provide some support for their argument.

Response: We would like to thank this reviewer for their time taken to review our manuscript. We now include additional information to emphasize that heterogeneity of stuttering is not a fact and is our hypothesis.

However, stuttering might not be heterogenous. For example, the "heterogeneity in results of both anatomical and functional human imaging studies," may reflect heterogeneity in the disorder, but may also reflect spurious findings due to small sample sizes, differing tasks, etc.

Response: We agree with the reviewer that the heterogeneity in the disorder may be due to sample sizes, differing tasks, etc. In the revised manuscript, we have removed any indication that this heterogeneity is a fact and we have stressed that it is our hypothesis.

To answer the authors' question, 30 is probably at the low end of a sufficient sample size, statistically. 30 is the extreme upper end of the range for stuttering studies (e.g., Fox et al., 1996 -> 10 AWS; Ingham et al., 2003 -> 10 AWS; Watkins et al., 2008 -> 12 AWS; Sommer et al., 2002 -> 15 AWS; Chang et al., 2009 -> 20 AWS). In an age of data dredging, it would be productive for researchers of stuttering to look at consistent findings vs. assuming all results are "right."

Response: We agree with the reviewer that the imaging data does not provide a coherent picture about this disorder. However, we have a different opinion about these results, and we do not believe that the results that do not provide a consistent finding are “wrong”. This heterogeneity in data could be because of different experimental methodologies. It also could be because of the heterogeneity of the disorder. Bottom line, dismissing peer reviewed, published data needs valid flaws in the design or analysis of data. We haven’t found any flaws in the cited papers in this manuscript and instead of dismissing the results, we believe considering stuttering as a heterogeneous disorder can explain this heterogeneity in imaging results.

My concern is that the authors are pitching testable hypotheses as facts. Thus, I think at the very least, the authors must soften their language to reflect that stuttering MAY be heterogenous in origin. The authors do this in some places, but not others, e.g.,

Response: We agree with the reviewer and in the revised manuscript have soften the language.

1)"As discussed above, known etiologies of stuttering (including genetics, excess dopamine, autoimmune response, and maybe others) are varied." The etiology is unknown.

Response: Thank you for the comment. The text of the revised manuscript now reads as, As discussed above, suggested etiologies of stuttering (including genetics, excess dopamine, autoimmune response, and maybe others) are varied.

2) "Stuttering is a heterogeneous disorder as etiology, phenotype, diagnostics, and responses to treatments differ among people with stuttering." Stuttering MAY be heterogenous.

Response: Thank you for the comment. The text of the revised manuscript now reads as, We hypothesize that stuttering could be considered as a heterogeneous disorder since etiology, phenotype, diagnostics, and responses to treatments differ among people with stuttering.

Comments from Reviewer 3:

I have carefully read the authors' response and the revised manuscript. The revised manuscript has minimal stylistic changes, and most comments and suggestions from Reviewer 1 and me have been dismissed.

Response: We would like to thank this reviewer for his/her time taken to review our manuscript. We respectfully disagree; as stated by Reviewer 1, “The authors have addressed my comments, except for their continued claim that stuttering IS heterogenous.” While this statement clearly shows that we have addressed the majority of Reviewer 1’s concerns, we do not understand why this Reviewer thinks otherwise! We did not dismiss any comments and provided a detailed response to all the comments raised by each reviewer in our 13 pages responses to reviewers’ comment and revised the manuscript thoroughly.

In their response to Reviewer 1 and me the authors keep referring to some "alternative view" on the pathophysiology of stuttering that I and the other reviewer seemingly hold. I do not know what the authors mean by the "alternative view"; using this argument to dismiss comments from your reviewers seems unproductive.

Response: We respectfully disagree. We do not believe that Reviewer 1 has an alternate view on the pathophysiology of stuttering and never mentioned that. Most of comments/criticism from this Reviewer lacks any reference. Therefore, it is hard for us to understand the reviewer’s point of view.

I still stand by my comments and invite the authors to revise their manuscript and present a more convincing argument supported by a thorough and comprehensive literature review (including data from children who stutter). Stuttering is a childhood disorder, children who stutter grow up to be adults who stutter (and develop learned behaviors in the process). Discussion of the etiology of stuttering based exclusively on adult data greatly limits the usability of the authors' perspective. Yet, the authors do not mention any limitations or caveats of their perspective.

Response: Our perspective is based on the peer-reviewed, scientifically evaluated published papers. As stated in our previous response, and according to one of the most respected and cited paper in stuttering field (cited by 494 published papers), “~ 90% of children who stutter recover spontaneously with or without intervention (Yairi and Ambrose, 2013).” Therefore, in our perspective, “we have mainly focused on adults who stutter (AWS).” We do not understand why this Reviewer insists on adding data from children who stutter. These data are outside the scope of the current perspective, and we have clearly stated that in the manuscript. In our opinion, we have a thorough review of the relevant literature for the purpose of this perspective. This Reviewer is insisting (without providing any reference) that adults who stutter “develop learned behaviors in the process” and reducing the symptoms of stuttering in adults to just “learned behaviors.” We responded to similar claim in our previous submission as well. Moreover, in light of the new research data (see below), this alternative view on pathophysiology of stuttering, i.e., the long-held dogma that stuttering is a behavioral or psychological condition, needs to be put to rest. Please see these recent articles (PMID: 34750604 and PMID: 33643015) that link stuttering to excess deposition of iron in the basal ganglia. These data strongly suggest that there are biological explanations for stuttering disorders. Also, please see these new data identifying new genes at the population level (https://doi.org/10.1016/j.xhgg.2021.100073 and PMID: 33894541). Again, these data further support the existence of a biological explanation for stuttering disorders. It has been erroneously suggested that developmental stuttering is caused by bad parenting, imitation, emotional trauma, or forcing a child that is left-handed to become righthanded. Some have even suggested that identifying a child as a person who stutters leads to more severe or persistent stuttering. We believe uncovering the genetic factors that underlie stuttering will highlight the molecular, cellular and circuit pathways involved in stuttering and inspire avenues of research for improved therapy/precision care. This is the first time that we are asked

to provide limitations or caveats for a perspective/hypothesis article! Respectfully, this manuscript is not a review paper or a research study. It is a hypothesis. We have provided a compelling support for the hypothesis that stuttering can be considered as a spectrum disorder. As we stated in our perspective, more experiments are needed to prove or disprove this hypothesis.

The authors state (lines 51-56) that "The Stuttering Spectrum Disorder (SSD) framework also improves the conventional model by grouping people who stutter based on their genetics and manifestation of stuttering, and therefore provides targeted treatment to the subgroups of adults/children who stutter. The SSD framework may facilitate recent efforts of precision medicine to provide personalized treatment plans to people with stuttering." I would like to see a detailed explanation supported by research references for each of the above claims: please explain how people who stutter should be grouped based on their genetics and manifestation of stuttering, please explain what treatments should be applied to what subgroups of people who stutter.

Response: The sentence referred by the reviewer is in the abstract. Below, we point the reviewer to the relevant sections in the main text of the previous submission, though we are not sure if the reviewer is implying to have all these information in the abstract! So far, there are three suggested causes for stuttering. We have discussed them in detail in lines 198-229 and 231-298. Based on the published data in peer-reviewed journals, for each of these groups, we have suggested specific treatments in lines: 256-258, 278-280, and 295-297. However, to respond to this comment, we have softened the language in the abstract.

It seems that the proposed stuttering heterogeneity mentioned in the manuscript has to do with the possible causes of stuttering. It could be related to four or five genetic mutations (what differences in brain circuits do these mutations cause?).

Response: “What differences in brain circuits do these mutations cause?” The answer is we do not know! Not the answer to this question is not understood in stuttering disorders, but we (and other neuroscientists) also do not know the answer for other psychiatric disorders, neurodevelopmental disorders, neurodegenerative disorders, sensory disorders, or neurological disorders that are related to one or multiple gene mutations! Therefore, we believe it is a significant flaw to request this information to be added to the manuscript. Regardless of the current lack of knowledge in this area, it is now established (by other researchers not us) that a mutation in lysosomal-trafficking pathway can lead to stuttering. We (and other group) are actively working to understand how a mild mutation in this gene may link to a motor control disorder that manifest stuttering phenotypes. However, to respond to this comment, we revised the manuscript and added more information regarding the recently discovered genes (i.e., ARNT2, SSUH2, CYRIA, ZMAT4) that might be associated with stuttering.

It could be related to a basal ganglia dysfunction (what genes are involved in this etiology?).

Response: Please see the text in previous submission (lines 213-214): “Stuttering is also proposed as basal ganglia disorder with an association to the elevated levels of presynaptic dopamine.” We did not propose any gene involvement in the basal ganglia, and we do not understand why the Review is asking for gene mutations in the basal ganglia! As we responded to this Reviewer before, genes and mutations in genes do not cause stuttering on their own, rather these mutations should modify brain circuits that are involved in complex motor behavior of speech production.

It could also be an autoimmune reaction to a streptococcal infection, which seemingly also leads to a basal ganglia dysfunction.

Response: Any disorders of motor control in mammals (not just humans) is related to the basal ganglia. However, the underlying mechanisms are different. So, if an autoimmune reaction causes stuttering (which is a motor control disorder), it is strongly related to the basal ganglia circuits [as it has been shown in other movement disorders (See PMID: 16225742; PMCID: PMC3398718; and others]. We should be clear that not all disorders related to the basal ganglia have similar mechanisms. This is actually our hypothesis that stuttering disorders have different underlying causes, even if they all be related to the basal ganglia.

Do these mechanisms represent three unique etiologies or are they related?

Response: We do not understand what the reviewer implying by this question. As discussed clearly in the manuscript (line 229-230), so far these are three proposed etiologies for stuttering. We view them as separate potential causes.

How is stuttering phenotype or "manifestation of stuttering" similar or different in people with the three proposed etiologies? Please provide a detailed discussion in the manuscript.

Response: There are no peer-reviewed published data about this question. If we had the information, it would be the fact that stuttering is a spectrum disorder. Again, this Reviewer asks for 'detailed discussion' on data that does not exist, and we strongly believe it is another significant flaw to request this information to be added to the manuscript. We have a whole section in the manuscript dedicated to the Inter-individual variations of stuttering phenotypes (lines 95-129). As we have mentioned several times in the manuscript, and even in the tile, this is our hypothesis that stuttering can be considered as a spectrum disorder. This hypothesis needs to be tested and we have clearly provided a road map on how to test this hypothesis (lines 313-226). Every reasonable scientist knows that a hypothesis could be true or could be wrong! In this perspective, we have provided evidence that argues in favor of this hypothesis. However, we have clearly stated in the conclusion section that future experiments in humans and animal models are needed.

The authors state that their perspective "improves the conventional model by grouping people who stutter based on their genetics and manifestation of stuttering". Which genetic mutations constitute a basis for a stuttering subgroup? If I were to plan a research study today, what criteria would I use to divide people who stutter into subgroups? In their response to my prior comment about this issue, the authors referred me to this paragraph: "With improved imaging techniques, nextgeneration DNA sequencing technologies, advanced electrophysiology, availability of animal models, and sophisticated deep learning algorithms, more studies should be done to define subtypes of the stuttering disorders under the umbrella of stuttering spectrum disorders (SSD). Combining genomic testing with next-generation analyses of imaging data will help clinicians to set criteria for each SSD sub-type and predict if a person with SSD would respond to certain therapies." Respectfully, the paragraph above provides little if any information on how to classify people who stutter into subgroups or define stuttering subtypes.

Response: Again, the sentence "improves the conventional model by grouping…" is from the abstract of the revised manuscript. We wonder why the two specific questions that the reviewer had, both were from the abstract! We have described our view on role of genetics in stuttering in details throughout the

manuscript. For the second part, we respectfully refer the reviewer to the same paragraph again. With improved imaging techniques, next-generation DNA sequencing technologies, advanced electrophysiology, availability of animal models, and sophisticated deep learning algorithms, more studies should be done to define subtypes of the stuttering disorders under the umbrella of stuttering spectrum disorders (SSD). Combining genomic testing with next-generation analyses of imaging data will help clinicians to set criteria for each SSD sub-type and predict if a person with SSD would respond to certain therapies. In this perspective, we hypothesized that stuttering is a spectrum disorder. Naturally, more experiments are needed to prove or disprove this hypothesis! If you want to plan a study on stuttering disorders today, you shouldn't have just one group consist of people who stutter and another group of control (i.e., fluent speakers). The researchers have to combine several other techniques such as advanced imaging and/or next-generation DNA sequencing technologies (actually these types of experiments are started to show up in the field; see PMID: 34859215 for example). In addition, animal research is also essential to define stuttering disorders at the cellular and circuit level (as these experiments are impossible to perform in humans at the moment). Therefore, as we have clearly stated in our manuscript, "more studies should be done to define subtypes of the stuttering disorders under the umbrella of stuttering spectrum disorders (SSD)." In summary, considering stuttering as an SSD is a hypothesis, and in this perspective article, we argue that considering stuttering as a spectrum disorder will allow us to perform more research to identify different subtypes of this disorder.

In closing, the SSD perspective is currently poorly defined and presents researchers with an impossible task of grouping people who stutter based on unspecified characteristics.

Response: We believe that this comment represents this Reviewer's point of view that the stuttering phenotype in adults who stutter is merely learned behaviors! The current literature (cited in this response and in the text) directly disprove this view. Respectfully, in this manuscript, we have postulated a hypothesis, and we provided a framework to test this hypothesis. We have clearly defined what we mean to consider stuttering as an SSD in the manuscript in the first line of Conclusions and future perspectives section: "The SSD refers to a broad range of conditions characterized by challenges with speech communication (i.e., stuttering phenotype). " If our framework is obsolete, then we would be greatly obliged if the Reviewer could provide reference(s) to a contemporary view of the key mechanism(s) underlying the pathophysiology of stuttering with modern therapeutic options in peer reviewed, scientifically published papers.

3rd Decision letter

Reference: CRNEUR-D-21-00027
Title: Stuttering as a spectrum disorder: a hypothesis
Journal: Current Research in Neurobiology

Dear Dr. Sheikhbahaei,

Thank you for submitting your manuscript to Current Research in Neurobiology.

I have completed my evaluation of your manuscript. The new reviewer and I have recommended reconsideration of your manuscript following major revision. I invite you to resubmit your manuscript after addressing the comments below. Please resubmit your revised manuscript by Apr 22, 2022.

When revising your manuscript, please consider all issues mentioned in the reviewers' comments carefully: please outline every change made in response to their comments and provide suitable rebuttals for any comments not addressed. Please note that your revised submission may need to be re-reviewed.

Current Research in Neurobiology values your contribution and I look forward to receiving your revised manuscript.

Kind regards,

Anna S Mitchell, Ph.D.
Editor in Chief
Current Research in Neurobiology

Comments from Editors and Reviewers:

Reviewer #4:

Overview: It is evident that this ms has gone through two revisions since its first submission. I am afraid that I will endorse yet another round of revision, which I know will frustrate the authors greatly and I apologize. However, I fully support the authors' hypothesis and think I can help point them in some helpful directions to make their point more compellingly.
I will not go down the article line by line conceptually, as there are two major issues that can be remedied. First is general organization, where topics (e.g., genetics, pharmacological response) are raised, and then raised again later. Some headings are meaningless (e.g., Stuttering Disorders in the middle of otherwise well-labeled topical paragraphs). It is really hard to follow the ms.
Second is the general narrowness of the possible arguments that can be made in support of the authors' position. They tend to use data with which they are most closely associated, such as pharmacological response and genetics. But there are numerous long-standing findings that should be used to argue the point: I will mention only a few to get the authors started. First, a well-known study by Ben Watson and colleagues showed two very clear subgroups of adults who stutter on linguistic ability and cerebral blood flow. Anne Foundas showed that only some boys who stutter showed atypical planum temporale symmetry across both hemispheres. Children who stutter show a fairly clear distinction between those with phonological disorder and those without (all the major labs: Illinois, Purdue, Syracuse/Vanderbilt). The same can probably be argued for language involvement in children (same sources, along with Ntourou meta-analysis). Clearly, some, but not all PWS show ADHD. Some, but not all, show early brain damage or infection (Alm and the authors' own work).
Thus, there are studies in abundance in any major text, such as Bloodstein (which does have a more recent edition than the one cited), which can easily be deployed to argue heterogeneity of PWS along any number of physiological as well as behavioral features (probably chapter by chapter in that large text). Response to treatment beyond pharmacological agents (behavioral therapies for adults and children, responses to altered auditory feedback) provide a plethora of other arguments. Critically, for these and other concepts, it is necessary for the authors to provide real numbers that relate back to

primary studies. Not numbers without references (such as the 80% remission rate, which is arguable), or, in contrast, arguments without specifics, such as numbers of PWS showing various profiles and carefully linked to contemporaneous primary reports (rather than using Bloodstein as a general reference or papers published in 1945). As a crude potential example that would be welcome and easily processed: "Watson et al. showed that their study group divided into 2 profiles; x% showed one response and y% showed another." Even the reports of identified genes supply general levels at which they were observed in the study participants, but which are not reported in this ms. In the interests of space, one could even table all of these concepts and variability across studies.
In contrast, some arguments for the endorsement of a spectrum or heterogeneity among PWS had me thinking argumentatively a bit. The notion that response to medications inherently implies heterogeneity or a spectrum disorder gave me pause for a moment - is that necessarily true? We know that not all individuals will respond in the same way to a single pharmacological intervention, but does that always imply that the underlying cause of the disorder is different, or that the symptoms vary along a spectrum? The logic here may require more explanation or documentation because I am not sure I see it.
I also would recommend that the authors seek a proof-reader, as there are numerous writing errors that interfere with reading and appreciation of the authors' viewpoint (to mention only a few of the many sentences I circled by line: 62 -considers AS A neurodevelopmental disorder?, 75- stuttering IS KNOWN for centuries 119 - stuttering DOES NOT caused by anxiety, 173 - normal SINCERITY of speech? A general observation is that articles (the) are sometimes used when not typically in native English, and lacking where they are.
I will stop here. I can see a lot of reasons to like the concept that the authors are pitching. Working backwards, if we started reporting some data differently, such as the number of PWS who show an atypical profile and those who do NOT, who respond to a treatment and those who do NOT, we would be well on our way to supporting the authors' contention, as well as their premise that customized treatments are the right way to go (who can argue this for MOST disorders?) Put another way, if the authors were proposing to INVESTIGATE the possibility that stuttering is a spectrum disorder and urging our studies and reporting to consider this notion, I'd think it was a slam dunk for most experts in our field to endorse.

3rd Author Response Letter

Response to comments from Editors and Reviewers:

Comments from Reviewer 4:

Overview: It is evident that this ms has gone through two revisions since its first submission. I am afraid that I will endorse yet another round of revision, which I know will frustrate the authors greatly and I apologize. However, I fully support the authors' hypothesis and think I can help point them in some helpful directions to make their point more compellingly.

Response: We would like to thank this reviewer for their time taken to review our manuscript and very positive assessment of our work. We now include additional information in our revised manuscript and provide detailed responses to all the comments raised.

I will not go down the article line by line conceptually, as there are two major issues that can be remedied. First is general organization, where topics (e.g., genetics, pharmacological response) are raised, and then raised again later. Some headings are meaningless (e.g., Stuttering Disorders in the middle of otherwise well-labeled topical paragraphs). It is really hard to follow the ms.

Response: We thank the reviewer for this comment. In the revised text, we have restructured the text to help the flow and to reduce the redundancies in the manuscript.

Second is the general narrowness of the possible arguments that can be made in support of the authors' position. They tend to use data with which they are most closely associated, such as pharmacological response and genetics.

Response: We thank the Reviewer for this comment. For clarification, our field recognized heterogeneity in stuttering with the published diagnostic criteria in DSM 5 differentiating childhood onset from other etiologies of stuttering. We expand beyond this already established heterogeneity by noting the differences beyond genetics and pharmacologic response. We outline existing, documented variability of stuttering phenotypes as well as the heterogeneous imaging data, variable responses to even nonpharmacological therapies and the expanded evidence lending to autoimmune, inflammatory responses. In synthesizing these growing data, we can outline at least 3 different etiologies or heterogenous foundations of stuttering--namely mutations in genes that are related to the lysosomal-trafficking pathway, disfunction of basal ganglia (also seen in the Reviewer comments regarding the high existence of comorbid other basal ganglia conditions like ADHD and OCD), and autoimmune reactions/inflammatory responses including association with migraine.

But there are numerous long-standing findings that should be used to argue the point: I will mention only a few to get the authors started. First, a well-known study by Ben Watson and colleagues showed two very clear subgroups of adults who stutter on linguistic ability and cerebral blood flow. Anne Foundas showed that only some boys who stutter showed atypical planum temporale symmetry across both hemispheres. Children who stutter show a fairly clear distinction between those with phonological disorder and those without (all the major labs: Illinois, Purdue, Syracuse/Vanderbilt). The same can probably be argued for language involvement in children (same sources, along with Ntourou meta-analysis). Clearly, some, but not all PWS show ADHD. Some, but not all, show early brain damage or infection (Alm and the authors' own work).

Response: Thank you for the comment and we will add such heterogeneity evidence as cited above. We agree with the Reviewer that we have not discussed all the existing data.

Thus, there are studies in abundance in any major text, such as Bloodstein (which does have a more recent edition than the one cited), which can easily be deployed to argue heterogeneity of PWS along any number of physiological as well as behavioral features (probably chapter by chapter in that large text).

Response: We believe cited studies in statistical and biological genetics, and data reported concerning brain structures and functions associated with sensory and motor speech, have demonstrated their tremendous potential for differentiating subgroups among adults who stutter. We value this comment and have added more data from primary sources to the manuscript.

Response to treatment beyond pharmacological agents (behavioral therapies for adults and children, responses to altered auditory feedback) provide a plethora of other arguments. Critically, for these and other concepts, it is necessary for the authors to provide real numbers that relate back to primary studies. Not numbers without references (such as the 80% remission rate, which is arguable), or, in contrast, arguments without specifics, such as numbers of PWS showing various profiles and carefully linked to contemporaneous primary reports (rather than using Bloodstein as a general reference or papers published in 1945). As a crude potential example that would be welcome and easily processed: "Watson et al. showed that their study group divided into 2 profiles; x% showed one response and y% showed another."

Response: Thank you for the comment. We like to highlight that in this perspective, we did not focus on pharmacological agents in our manuscript rather mentioned that it is an available treatment

option. We have discussed response to treatments beyond pharmacological agents in a great detail in the manuscript, even more than pharmacological treatments (specifically, pharmacological treatments are mentioned in lines 191-196 and other therapies are mentioned in lines165-189). Critically, double-blind placebo clinical trials (which is a gold standard in evaluating any treatment) are lacking in published data from speech and cognitive therapies. It is difficult to really establish treatment effects in the vast majority of therapeutic trials in stuttering as the designs are not usually blinded and do not take into account the natural spontaneous remission (in studies in pediatric populations). It has also been established that the metrics utilized to measure clinical response in stuttering have not been validated for repeated clinically meaningful effects over time (e.g., the SSI and OASES) especially given the natural variability in fluency. In addition, the number of participants in each group [experimental (i.e., people who stutter) and control (i.e., people who do not stutter)] in the peerreviewed published papers are small. Therefore, we are not sure providing effectiveness of treatments as a percentage of total people would be an appropriate way to report the data. Since our manuscript is a hypothesis paper, we believe existence of even a small fraction of people who do not respond to a treatment in each of these studies is indicative of existence of subgroups in the pathophysiology of stuttering. All the data presented in the manuscript is based on the published, peer-reviews papers. In that regard, we do not understand the Reviewer's reference to "80% remission rate," as we do not have such a phrase in our manuscript. However, we have double checked the text to make sure that all the references are provided. We referenced to Brown 1945 as an example that the inter-individual variability is not new and has been reported before. We believe it is important to keep it in mind that there are at least ~ 80 years of data on this topic. In our opinion, considering stuttering as a spectrum disorder can explain this heterogeneity in symptoms. However, to respond to this comment of the reviewer, we added more data from the primary literature to the manuscript.

Even the reports of identified genes supply general levels at which they were observed in the study participants, but which are not reported in this ms. In the interests of space, one could even table all of these concepts and variability across studies.

Response: We do not understand the Reviewer's comment about "the reports of identified genes supply general levels at which they were observed in the study participants". We have stated in the manuscript that the discovered genes (GNPTAB and others) account for up to 20% of the people who stutter (based on the existing, published data).

In contrast, some arguments for the endorsement of a spectrum or heterogeneity among PWS had me thinking argumentatively a bit. The notion that response to medications inherently implies heterogeneity or a spectrum disorder gave me pause for a moment - is that necessarily true? We know that not all individuals will respond in the same way to a single pharmacological intervention, but does that always imply that the underlying cause of the disorder is different, or that the symptoms vary along a spectrum? The logic here may require more explanation or documentation because I am not sure I see it.

Response: Thank you for the comment. To illustrate our point, let's consider sore throat (pharyngitis) as an example which have different causes. If we just want to treat based on just symptoms and do not perform any test to know if the cause is viral, bacterial, or allergy, we cannot provide effective treatments for a significant percentage of patients. Therefore, some patients will not respond to the treatment. This heterogeneity in responses to a treatment, strongly suggests that there are different etiologies of sore throat (pharyngitis). We believe the same logic also apply here. The fact that a subgroup of people who stutter respond to a treatment (speech therapy and/or pharmacology therapy) strongly suggests that other underlying etiologies exist. This model of backing into etiology based on response to treatment is the primary lead in research with other neuropsychiatric

conditions such as obsessive-compulsive disorder (OCD), Tourette's Syndrome, and ADHD which of note, have high co-morbidity with stuttering.

I also would recommend that the authors seek a proof-reader, as there are numerous writing errors that interfere with reading and appreciation of the authors' viewpoint (to mention only a few of the many sentences I circled by line: 62 -considers AS A neurodevelopmental disorder?, 75- stuttering IS KNOWN for centuries 119 - stuttering DOES NOT caused by anxiety, 173 - normal SINCERITY of speech? A general observation is that articles (the) are sometimes used when not typically in native English, and lacking where they are.

Response: Although the manuscript was edited by the National Institutes of Health Editing Service (by Brigit S. Sullivan, MLS, a Biomedical Librarian at the National Institutes of Health Library; stated in the cover page of the submission), we asked other professional editors to take another look at the manuscript.

I will stop here. I can see a lot of reasons to like the concept that the authors are pitching. Working backwards, if we started reporting some data differently, such as the number of PWS who show an atypical profile and those who do NOT, who respond to a treatment and those who do NOT, we would be well on our way to supporting the authors' contention, as well as their premise that customized treatments are the right way to go (who can argue this for MOST disorders?) Put another way, if the authors were proposing to INVESTIGATE the possibility that stuttering is a spectrum disorder and urging our studies and reporting to consider this notion, I'd think it was a slam dunk for most experts in our field to endorse.

Response: Thank you for the comment. In this manuscript we are proposing that stuttering can be considered as a spectrum disorder. We have also provided a framework to test this hypothesis. Based on this comment of the reviewer, we now encourage other researchers to consider this possibility and test this hypothesis in a scientific way. It is important to note that researchers started to consider subtyping in other neurodevelopmental disorders (see PMCID: PMC4766622 for instance). To illustrate our point, let's consider the sore throat (pharyngitis) example again. This is a symptom and can occurs due to different types of infections (bacterial or viral). It is important to know the cause so that we (as care providers) can provide the "right treatment." The future of therapeutics resides in personalized medicine. Of note, breast cancer therapy is a fantastic example. For years, all breast cancers were treated the same. Now, with understanding of different sub-types based on genetic profiles and staging, even though the clinical symptoms may appear similar, therapies are based upon individualized profiles. People who stutter show heterogeneity in their clinical symptoms, neuroimaging, responses to therapies, and differences in genetic profiles. Similar to our advances in oncology and the growing field of personalized medicine, evidence strongly suggests now that stuttering may arise from multiple etiologies and is thus a heterogenous condition necessitating individualized, personalized therapies. Understanding more about this disorder (by utilizing, for example, advanced neuroimaging, genetic profiling including pharmacogenomics, and the utilization of animal models to translate to human) will enable our field to optimize therapies for those who stutter through personalized treatment. The data exists warranting further research in elucidating the specifics behind the heterogeneity of stuttering.

4th Decision letter

Reference: CRNEUR-D-21-00027
Title: Stuttering as a spectrum disorder: a hypothesis
Journal: Current Research in Neurobiology

Dear Dr. Sheikhbahaei,

Thank you for submitting your manuscript to Current Research in Neurobiology.

I have completed my evaluation of your manuscript. The reviewers recommend reconsideration of your manuscript following revision. I invite you to resubmit your manuscript after addressing the comments below. Please resubmit your revised manuscript by Oct 31, 2022.

When revising your manuscript, please consider all issues mentioned in the reviewers' comments carefully: please outline every change made in response to their comments and provide suitable rebuttals for any comments not addressed. Please note that your revised submission may need to be re-reviewed.

Current Research in Neurobiology values your contribution and I look forward to receiving your revised manuscript.

Kind regards,

Anna S Mitchell, Ph.D.
Editor in Chief
Current Research in Neurobiology

Comments from Editors and Reviewers:

Unfortunately there is only one review with this revision as the other reviewer has declined to provide comments. I have reviewed the manuscript as well and agree with the comments of the reviewer. Please can you carefully address the comments of this one reviewer and seek out the help of a colleague to review your responses and ensure there is clarity in your edits.

Reviewer #4:

The authors have been fairly responsive to my concerns, although the ms, for all its revisions, still seems uneven. The argument for classifying stuttering as a spectrum disorder is made over and over again, making the ms seem repetitive, while some of the arguments still appear disjunct, although the flow has improved. For example, the notion that behaviors are heterogeneous is made in more than one place, still. And the citations and level of discussion is very weak, given how much of early work in the field went to great pains to describe individual profiles.
The quality of references for some of the assertions is very uneven: citations range from primary research, as would be appropriate in this case, to superficial (e.g., updates for generalist physicians), to

strangely selected (why Gerlach's fascinating work on the economic consequences of stuttering is referenced to substantiate access to treatments is very unclear).
Now that the article is somewhat easier to track (I really had so much trouble with the logic the first time around that I couldn't even identify some of my concerns easily), the decision to ground the arguments entirely in the adult "literature" seems either inappropriate or misguided. For one thing, lots of the citations inside the article are to children who stutter, not adults, so that would need to be rectified. But I think it actually weakens the authors' assertions, since a huge issue in treatment of childhood stuttering is the remission rate. It's high, but we have yet to determine which kids will recover, just some statistical predictors that are not sufficiently strong to classify individual children. So remission itself clearly leads to two subtypes: those who do and do not recover. Much of the physiology discussed, and a good deal of the genetics covered in the ms. seem to play some role in this. Thus, limiting the scope of argumentation to adults seems to miss the interwoven complexity of things that sort the children as well as the adults. It would seem easier to include them to strengthen the case than go through and re-evaluate the mixed bag of citations in text (some of which remain incomplete).
Finally, but not in terms of where it needs to be discussed (early would be my suggestion) I think the whole notion of what it takes to defend a disorder as spectrum-like needs to be strengthened. Some level of heterogeneity of symptoms or response to treatment do not alone, in my opinion do this. Is there not some definition? Working from there might superimpose the order I am seeking, since some concepts, such as where in utterances people stutter (tip, guys, it differs from language to language, too!), or the role of iron, do not appear to fit anywhere comfortably in the set of crafted arguments. The ms appears organized around a small set of potential etiologies (genetics, basal ganglia disfunction, etc.) rather than around how the facts of stuttering square with what we usually expect from a syndrome.
As a frequent book and journal editor, I gave up my request to have someone edit the document more carefully, and just made an annotated version that I am conveying to the current editor. The comments unblind me, but hopefully the authors will understand that I do agree with their logic, if not the presentation currently in front of me. I do think it's just about there, but I wouldn't want the result to have weaknesses that are relatively easy to address.

4th Author Response Letter

Response to comments from Editors and Reviewers:

Comments from Reviewer 4:

The authors have been fairly responsive to my concerns, although the ms, for all its revisions, still seems uneven. The argument for classifying stuttering as a spectrum disorder is made over and over again, making the ms seem repetitive, while some of the arguments still appear disjunct, although the flow has improved. For example, the notion that behaviors are heterogeneous is made in more than one place, still.

Response: We would like to thank this reviewer for the time taken to review our manuscript. In the manuscript, we have provided data in different sections that *strongly* support our central hypothesis: stuttering is a heterogeneous disorder, and likewise, the underlying mechanisms are heterogeneous too. In this regard and to prevent any misinterpretation, we employed this writing style to convey our message clearly.

And the citations and level of discussion is very weak, given how much of early work in the field went to great pains to describe individual profiles.

Response: We like to emphasize again that his manuscript is not a review paper or a research study but a perspective paper; therefore, in our opinion, it is not necessary to summarize *all* the early work in this manuscript, as other great resources are available for that purpose. We also like to note that describing "individual profiles" of people who stutter is outside the scope of the current manuscript. In this manuscript, we used existing data to support our hypothesis that stuttering is a heterogeneous disorder. In addition, the manuscript is submitted under the "Hypotheses & Intersections" category to the **CRNEUR journal**. In this setting, based on the peer-reviewed, scientifically evaluated published papers, we have provided compelling support for the hypothesis that stuttering can be considered a spectrum disorder. As we stated in our manuscript, more experiments are needed to prove or disprove this hypothesis.

The quality of references for some of the assertions is very uneven: citations range from primary research, as would be appropriate in this case, to superficial (e.g., updates for generalist physicians), to strangely selected (why Gerlach's fascinating work on the economic consequences of stuttering is referenced to substantiate access to treatments is very unclear).

Response: It seems that the Reviewer is referring to this reference as a superficial: *Perez, H. R., and Stoeckle, J. H. (2016). Stuttering: Clinical and research update. Can Fam Physician 62, 479–484*. While the criteria for such an article are unclear, this referenced article is published in the *Canadian Family Physician's* journal (with an Impact Factor of 3.275). It has been cited 66 times in 6 years! This reference was used in line 120 when we described stuttering symptoms.

We also beleive the Reviewer referring to this sentence in the manuscript for the "strangely selected" reference: "*People who stutter may struggle with a lower quality of life, face educational and occupation barriers, and be denied access to quality treatment plans (Koedoot et al., 2011; Gerlach et al., 2018).*" With more careful reading, we believe it would be apparent that Gerlach et al. is provided as a reference for the "lower quality of life, face educational and occupation barriers" section of the sentence. In the revised text, we have updated the text to indicate this more clearly.

Now that the article is somewhat easier to track (I really had so much trouble with the logic the first time around that I couldn't even identify some of my concerns easily), the decision to ground the arguments entirely in the adult "literature" seems either inappropriate or misguided. For one thing, lots of the citations inside the article are to children who stutter, not adults, so that would need to be rectified.

Response: We thank the reviewer for this comment; however, we respectfully disagree that focusing on adults who stutter is "inappropriate or misguided." As we clearly stated in the manuscript, "*the majority of children who stutter (CWS) recover through an unknown mechanism by their adolescence (Chow & Chang, 2017; Dworzynski et al., 2007; Yairi & Ambrose, 2013; Smith & Weber, 2016).*" The existing experimental data on potential causes of developmental stuttering disorder(s) in children are minimal, and in our opinion, no hypothesis can be made from those limited published data. On the other hand, in adults who stutter, in our opinion, sufficient data exist to formulate a hypothesis regarding the heterogeneity of stuttering. We also like to note that we have referred to stuttering disorders as "childhood-onset fluency disorder" (stated in the highlights, abstract, and the first line of the introduction). In our opinion, the term (i.e., childhood-onset fluency disorder) indicates that the disorder/condition has started from childhood (i.e., any underlying mechanism(s) should have started

from childhood). In this regard, since the disorder started in childhood, we have cited papers that described the disorder in children (for example, four papers have been cited for that one sentence mentioned above). We agree that a new framework to describe stuttering in children who stutter might be necessary, but it is outside the scope of our current perspective.

However, to respond to this comment, we have added a complete section on the viability of data in children who stutter [Lines 362-404] to argue that the SSD can potentially be expanded to the CWS as well.

But I think it actually weakens the authors' assertions, since a huge issue in treatment of childhood stuttering is the remission rate. It's high, but we have yet to determine which kids will recover, just some statistical predictors that are not sufficiently strong to classify individual children. So remission itself clearly leads to two subtypes: those who do and do not recover. Much of the physiology discussed, and a good deal of the genetics covered in the ms. seem to play some role in this. Thus, limiting the scope of argumentation to adults seems to miss the interwoven complexity of things that sort the children as well as the adults. It would seem easier to include them to strengthen the case than go through and re-evaluate the mixed bag of citations in text (some of which remain incomplete).

Response: We thank the Reviewer for this comment. We (and others) have stated that the recovery mechanism(s) of stuttering in children is not known; likewise, the mechanism(s) of remission are not known yet. In addition, the experimental data on genetics in children is limited, and we do not feel comfortable proposing a hypothesis based on unknown mechanisms and limited data.

We also like to point out that "issue[s] in treatment of childhood stuttering" is outside this manuscript's scope. As stated in the manuscript, speech and cognitive therapies are first-line treatments, and the response to these treatments is also variable. It is plausible that based on the underlying causes, the recovery/remission might be different. In this manuscript, we have focused on the causes of stuttering. In fact, we firmly believe that by knowing more about the various mechanisms that might lead to stuttering, one can propose novel treatments for each cause. Over time, it will undoubtedly help with the "issue in the treatment of childhood stuttering."

Finally, but not in terms of where it needs to be discussed (early would be my suggestion) I think the whole notion of what it takes to defend a disorder as spectrum-like needs to be strengthened.

Response: We agree with the Reviewer that more information is needed to define a disorder as a spectrum, and that is the reason that this manuscript is submitted as a <u>hypothesis</u>: we have posited that based on the existing data (including symptoms, imaging, and responses to treatments), the heterogeneity of symptoms in people who stutter strongly <u>*suggest*</u> that the underlying causes <u>*may be*</u> different as well. As we have stated in the manuscript, “more studies *should be done*” to prove or disprove this hypothesis.

Some level of heterogeneity of symptoms or response to treatment do not alone, in my opinion do this. Is there not some definition? Working from there might superimpose the order I am seeking, since some concepts, such as where in utterances people stutter (tip, guys, it differs from language to language, too!), or the role of iron, do not appear to fit anywhere comfortably in the set of crafted arguments.

Response: We respectfully disagree. We argued that heterogeneity of data regarding etiology, phenotype, diagnostics, and responses to treatments in people who stutter exists. Based on these data, we hypothesized that stuttering could be considered a spectrum disorder. Respectfully, this manuscript is not about the characteristics of stuttering in people who stutter. The whole argument is that we should not just focus on the phenotype (i.e., the stuttering in this case) to define this disorder!

Regarding iron, data from two independent studies *suggested* that elevated iron might have a role in the pathophysiology of stuttering. Interestingly, both data pointed toward elevated iron leves in the basal ganglia, further strengthening the hypothesis that a deficit in basal ganglia function may lead to stuttering disorder. Although we believe that these data fit comfortably in the basal ganglia hypothesis, in the manuscript, we have stated that "*further experiments are required to define a mechanism by which this elevated iron could cause the stuttering disorder*."

The ms appears organized around a small set of potential etiologies (genetics, basal ganglia disfunction, etc.) rather than around how the facts of stuttering square with what we usually expect from a syndrome.

Response: Only a small set of potential etiologies for stuttering are proposed in the peer-reviewed and published literature. In this perspective, we have stated, "*Recent experimental data point toward involvement of at least three potential causes for stuttering,"* suggesting that more etiologies are yet to be discovered. In addition, many lines of evidence from different labs support the involvement of genetic substrates and/or basal ganglia dysfunction in the pathophysiology of stuttering. In this regard, we do not agree with the Reviewer to consider stuttering as a syndrome; in fact, in our perspective, we have hypothesized that stuttering can be considered a spectrum disorder.

As a frequent book and journal editor, I gave up my request to have someone edit the document more carefully, and just made an annotated version that I am conveying to the current editor. The comments unblind me, but hopefully the authors will understand that I do agree with their logic, if not the presentation currently in front of me. I do think it's just about there, but I wouldn't want the result to have weaknesses that are relatively easy to address.

Response: We thank the reviewer for the sending an annotated version, however, we did not receive such a document. We like to note that two different professional editors have edited our manuscript at the National Institutes of Health. Again, we remain committed to addressing any additional specific concerns the reviewer might raise, as well as correcting any inadvertent specific errors. Thank you for the positive evaluation of our manuscript.

Accept Letter

Dear Dr. Sheikhbahaei,

Thank you for submitting your manuscript to Current Research in Neurobiology.

I am pleased to inform you that your revised manuscript has been accepted for publication.
Congratulations.

Your accepted manuscript will now be transferred to our production department. We will create a proof

which you will be asked to check, and you will also be asked to complete a number of online forms required for publication. If we need additional information from you during the production process, we will contact you directly.

We appreciate and value your contribution to Current Research in Neurobiology. We regularly invite authors of recently published manuscript to participate in the peer review process. If you were not already part of the journal's reviewer pool, you have now been added to it. We look forward to your continued participation in our journal, and we hope you will consider us again for future submissions.

CRNEUR aims to be a unique, community-led journal, as highlighted in the Editorial Introduction. As part of this vision, we will be regularly seeking input from the scientific community and encourage you and your co-authors to take the survey.

We would also like to invite you to take part in our CRNEUR Author Question & Answer (Q&A), which could get published alongside your article and help to promote it. We suspect you might have an interesting story of perseverance or team work that was required for the research study to complete, or a diversity of perspectives that you might share, as a way of inspiring others about neuroscience.

Kind regards,

Anna S Mitchell, Ph.D.
Editor in Chief
Current Research in Neurobiology

-------- *End of Review Comments* --------
